# Advancing the Landscape of RNAi Nanotherapeutics for Ischemic Heart Disease

**DOI:** 10.1002/adma.202523005

**Published:** 2026-02-17

**Authors:** Han Gao, Da Pan, Hélder A. Santos

**Affiliations:** ^1^ Department of Pharmacology School of Medicine Southeast University Nanjing Jiangsu China; ^2^ Department of Nutrition and Food Hygiene School of Public Health Key Laboratory of Environmental Medicine and Engineering of Ministry of Education Southeast University Nanjing Jiangsu China; ^3^ Department of Biomaterials and Biomedical Technology The Personalized Medicine Research Institute (PRECISION) University Medical Center Groningen (UMCG) University of Groningen Groningen The Netherlands

**Keywords:** drug delivery, gene therapy, ischemic heart disease, nanomedicine, RNAi therapeutics

## Abstract

Ischemic heart disease (IHD) remains the foremost cause of mortality worldwide, characterized by extensive myocardial injury following infarction with limited regenerative capacity of the adult mammalian heart. RNA interference (RNAi) nanotechnology is revolutionizing cardiac therapy by leveraging the sequence‐oriented gene regulation that can overcome the obstacles hindering conventional therapeutic approaches. Here, we outline the evolving landscape of RNAi nanomedicine for cardiac repair, with an emphasis on therapeutic advances in myocardial infarction and atherosclerosis. We further discuss design considerations for constructing effective nanosystems, underscoring the critical principles by bridging mechanistic insights with material engineering. Finally, we delineate the translational pathway from bench to bedside, detailing current challenges and highlighting prospective opportunities for advancing RNAi therapeutics in cardiovascular therapy.

## Graphical abstract

RNA interference (RNAi) nanomedicine revolutionizes treatment regimens for ischemic heart diseases by enabling tailored, sequence‐anchored gene regulation. This review highlights the recent advances in nanotechnology‐driven RNAi therapeutics for myocardial ischemia and discusses the key design principles that govern efficient delivery, providing critical insights into the rational design of next‐generation nanoplatforms for cardiac repair.

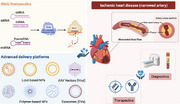

**Abbreviations**
AAV: adeno‐associated virus
AAV9: adeno‐associated virus 9
AgNPs: silver nanoparticles
AHP: acute hepatic porphyria
AI: artificial intelligence
AS: atherosclerosis
ASOs: antisense oligonucleotides
AT: atorvastatin
apoE: apolipoprotein E
BCL2L11: Bcl‐2‐like protein 11
BO: bayesian optimization
CaMKIIγ: Ca^2+^/calmodulin‐dependent protein kinase γ
CDCs: cardiosphere‐derived cells
CGMD: coarse‐grained molecular dynamics
CHD: coronary heart disease
CMs: cardiomyocytes
CMP: cardiomyocyte‐specific binding peptide
cRGD: cyclic cRGDfK peptide
CR3: complement receptor 3
CRD: carbohydrate recognition domain
CVDs: cardiovascular diseases
Cy3: cyanine3
Cy5.5: cyanine5.5
DFT: density functional theory
DPD: dissipative particle dynamics
dsRNA: double‐stranded RNA
DSPE: 1,2‐distearoyl‐sn‐glycero‐3‐phosphorylethanolamine
EE: early endosome
ESC: european society of cardiology
EV: extracellular vesicle
FACS: fluorescence‐activated cell sorting
FDA: food and drug administration
GalNAc: triantennary N‐acetylgalactosamine carbohydrates
HA: hyaluronic acid
HA‐DOPE: HA‐dioleoylphosphatidylethanolamine
HDR: high‐dynamic‐range
hESCs: human embryonic stem cells
HF: heart failure
HFpEF: heart failure with preserved ejection fraction
Hmmr: hyaluronan‐mediated motility receptor
i.v.: intravenous
IHD: ischemic heart disease
iPSCs: induced pluripotent stem cells
iPSC‐CMs: induced pluripotent stem‐cell‐derived cardiomyocytes
IR: ischemia reperfusion
KDR: kinase insert domain receptor
LDL: low‐density lipoprotein
LE: late endosome
LECs: lymphatic endothelial cells
LNPs: lipid nanoparticles
MACE: major adverse cardiovascular events
MAPK: mitogen‐activated protein kinase
MD: molecular dynamics
miRNAs: microRNAs
miR‐21: microRNA‐21
miRNPs: ribonucleoprotein complexes contain numerous miRNAs
ML: machine learning
MMMs: mixed matrix membranes
MOF: metal‐organic framework
MPS: mononuclear phagocyte system
mRNA: messenger RNA
MT: microtubules
MW: molecular weight
Nabs: neutralizing antibodies
NF‐κB: nuclear factor‐κB
NLRP1: NLR family pyrin domain containing 1
OLE: open‐label extension
oxLDL: oxidized low‐density lipoprotein
PAMAM: poly(amidoamine)
PBAE: poly(β‐amino ester)
PC: poly(l‐lysine)‐cis‐aconitic acid
PCSK9: proprotein convertase subtilisin kexin 9
PEG: polyethylene glycol
PEI: polyethylenimine
PH1: primary type 1 hyperoxaluria
PI3K: phosphoinositide 3‐kinase
piRNAs: Piwi‐interacting RNAs
PLGA: poly(lactic‐co‐glycolic) acid
RAFT: reversible addition‐fragmentation chain transfer
RISC: RNA‐induced silencing complex
RNAi: RNA interference
s.c.: subcutaneous
siRNAs: small interfering RNAs
SMCs: smooth muscle cells
SOCS3: suppressor of cytokine signaling 3
SQM: semiempirical quantum mechanics
sRNA: small RNA
sRNAs: small RNAs
TGF‐β: transforming growth factor‐beta
TLR4: toll‐like receptor 4
TPOT: tree‐based pipeline optimization tool
TREM2: triggering receptor expressed on myeloid cells 2
VECs: vascular endothelial cells
VSMCs: vascular smooth muscle cells
β‐glucans: beta‐glucans.

## 1 Introduction

Cardiovascular diseases (CVDs) encompass a broad spectrum of disorders involving the heart and blood vessels, with ischemic heart disease (IHD) representing the most prevalent and life‐threatening condition [1]. Despite significant advances made over the past decade, the overall prognosis for CVDs remains poor, highlighting the imperative to explore innovative strategies for prevention and personalized medication [2, 3]. According to the latest updates of guidelines from the European Society of Cardiology (ESC), the recommended management for acute coronary conditions, such as myocardial infarction (MI), includes pharmacotherapy and an immediate reperfusion strategy within 24 h post‐ischemia [4]. Yet, due to the intricate nature of cardiac vasculature, alterations across the arterio‐venous axis, cardiomyocyte loss during ischemia, and other factors, there remains a notable absence of specific pharmacological interventions recommended for distinct cardiac conditions, such as type 2 MI [4, 5].

Nanotechnology has catalyzed a paradigm shift in our understanding of molecular biology under physiological and pathological conditions. A defining milestone was the clinical approval of non‐viral vector‐formulated gene therapy in 2018 [6, 7]. Following this, the COVID‐19 pandemic witnessed the clinical efficacy of lipid nanoparticles (LNPs) for targeted messenger RNA (mRNA) vaccination [8]. Nevertheless, in the context of cardiovascular diseases, clinical translation of comparable gene therapies remains limited; some trials were ended by unsatisfactory outcomes than expected, underscoring the need for advancing the design of delivery materials [9]. Moreover, elucidating the interface between nanomaterials and biological systems, underlying mechanisms of cellular uptake and intracellular trafficking are essential for overcoming the current hurdles and improving efficacy in clinics [10].

RNA interference (RNAi) therapeutics represent a novel class of pharmaceutical drugs for gene therapy, differing from traditional therapeutic approaches, where RNAi applies to all classes of molecular targets by interfering with the target mRNA via the RNA‐induced silencing complex (RISC) machinery [11, 12]. Consequently, RNAi therapeutics hold great potential for personalized medicine, especially since the first RNAi drug approved by the US Food and Drug Administration (FDA), which opened a new frontier in the field of gene therapy [13, 14]. Following this, the clinical landscape of RNAi therapeutics in the cardiovascular domain has evolved rapidly, although most of them are undergoing clinical trials or are in preclinical phases. A key milestone was the landmark approval of Inclisiran in 2021, representing the first GalNAc‐conjugated siRNA therapy for the management of hypercholesterolemia [15]. More recently, the latest report from the HELIOS‐B trial demonstrated compelling clinical efficacy of vutrisiran on cardiac structure and function, further expanding the utility of RNAi therapeutics to the treatment of transthyretin amyloidosis with cardiomyopathy (ATTR‐CM), heralding a transformative era of ‘programmable’ cardiovascular medicine. For future endeavors, unraveling molecular pathways involved in pathogenesis, refining targeting specificity of delivery systems, and achieving controlled systemic release of RNAi payloads are essential to enable the next generation of clinical breakthroughs.

In this review, we summarize the current landscape of RNAi therapeutics in ischemic heart diseases, particularly the applications in myocardial infarction and atherosclerosis. We further emphasize the general requirements for developing genetic delivery vehicles and discuss critical factors in constructing the delivery framework. Subsequently, we provide a brief overview of recent advances in utilizing nanoplatforms for enabling precise and targeted delivery of genetic cargos. Finally, we introduce emerging strategies to unravel nano‐bio interactions and the underlying mechanisms governing intracellular trafficking.

## 2 Principles and Modalities of RNAi Therapeutics

**FIGURE 1:**
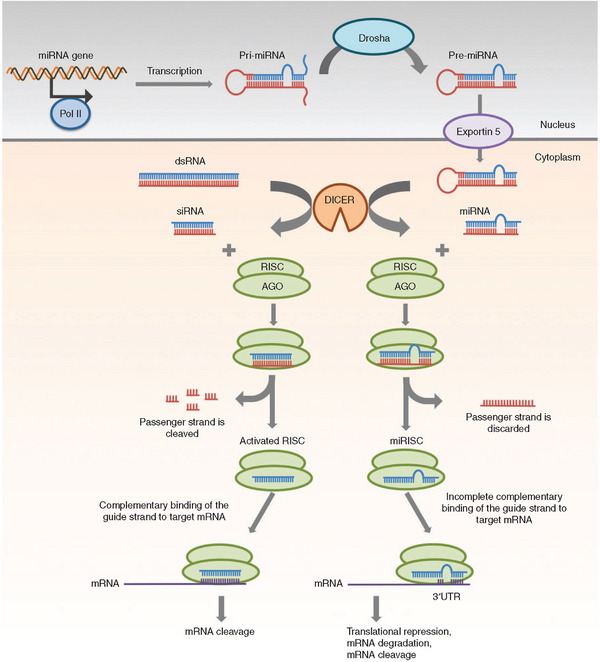
Gene silencing mechanisms of siRNA and miRNA. siRNA: dsRNA (either transcribed or artificially introduced) is processed by Dicer into siRNA that is loaded into the RISC. AGO2, which is a component of RISC, cleaves the passenger strand of siRNA. The guide strand then guides the active RISC to the target mRNA. The full complementary binding between the guide strand of siRNA and the target mRNA leads to the cleavage of mRNA. miRNA: Transcription of the miRNA gene is carried out by RNA polymerase II in the nucleus to give pri‐miRNA, which is then cleaved by Drosha to form pre‐miRNA. The pre‐miRNA is transported by Exportin 5 to the cytoplasm, where it is processed by Dicer into miRNA. The miRNA is loaded into the RISC, where the passenger strand is discarded, and the miRISC is guided by the remaining guide strand to the target mRNA through partially complementary binding. The target mRNA is inhibited via translational repression, degradation, or cleavage. Reproduced with permission [21]. Copyright 2015, Elsevier B.V. Reproduced under the CC BY‐NC‐ND 4.0 License.

RNA interference is the technique involving the introduction of exogenous double‐stranded RNA (dsRNA) molecules to disrupt the function of an endogenous gene, which was originally validated and demonstrated superior efficacy in experimental animals and their progeny [16, 17]. In particular, the accomplishment of gene silencing was shown to be accompanied by ∼20–30 nucleotide small RNA (sRNA) molecules to match the target sequences via base‐pairing interactions [18]. Depending on their origins, transcriptomic mechanisms, chemical structures, and biological functions, small RNAs (sRNAs) can be classified into three main categories: small interfering RNAs (siRNAs), microRNAs (miRNAs), and piwi‐interacting RNAs (piRNAs) [18, 19]. Although sharing the common role as sequence‐specific posttranscriptional regulators, siRNAs and miRNAs are distinct molecules in terms of biogenesis and the machinery working scheme for gene silencing, as illustrated in Figure 1 [20].

### 2.1 MicroRNAs (miRNAs)

Endogenous miRNAs are initially processed from genomic transcripts that contain complementary or near‐complementary 20‐ to 50‐base‐pair inverted repeats, which fold back on themselves to form double‐stranded RNA (dsRNA) hairpins [22]. Many miRNAs are shown to be highly distributed in eukaryotic organisms with distinctive expression patterns. For example, following a large sequencing analysis, a cluster of miRNAs accounts for maintaining the cardiac function under physiological conditions, such as miR‐16 and miR‐27b [23, 24]. Moreover, emerging studies identified miRNAs as critical regulators involved in cardiac remodeling [23, 24, 25]. When the heart is exposed to external stressors or acute triggers, the onset of sterile inflammation promotes the circulation of miRNAs, which can block translation or induce degradation of messenger RNA (mRNA) and thereby contribute to the pathophysiological consequences of ischemic myocardium [26]. In contrast, instead of increased expression, some miRNAs showed beneficial effects upon expression elimination. For instance, higher incidences of MI and heart failure (HF) were shown in aging populations. By adopting a high‐throughput fluorescence‐activated cell sorting (FACS)‐based method, researchers identified miR‐22 as an abundant and strong inhibitor of the cardiac autophagy process [27].

Upon pharmaceutical inhibition, decreased miR‐22 levels activated cardiac autophagy, inhibiting cellular hypertrophy and improving cardiac function [27]. In addition to serving as therapeutic targets, circulating miRNAs can be considered potential diagnostic biomarkers for CVDs. In patients with acute ST‐elevation MI, four miRNAs increased abruptly in plasma with a peak within 12 h, including miR‐1, miR‐133a, miR‐208b, and miR‐499‐5p [28]. In a recent study by Maria et al., the levels of microRNA‐21 (miR‐21) in the peripheral blood monocytes proved to be significantly associated with the occurrence of heart failure (HF) with preserved ejection fraction (HFpEF), underscoring its diagnostic value in HF [29]. Similar studies were conducted in this field, contributing to discovering specific miRNAs involved in myocardial failure [30, 31, 32, 33].

Altogether, these findings suggest the critical role of miRNAs in the diagnosis and treatment of CVDs; further studies investigating the underlying mechanisms are warranted to provide more insights before translation in clinical practice.

### 2.2 Small Interfering RNAs (siRNAs)

Similar to miRNAs, siRNAs are another type of genetic tool that can interfere with specific mRNAs for cleavage or translational repression [20]. Nevertheless, the assembly into RNA silencing effector complexes differs in two biological molecules; an siRNA‐containing effector complex is commonly referred to as a RISC, whereas ribonucleoprotein complexes that contain numerous miRNAs (miRNPs) are referred to as one of miRNAs‐based effector complexes [22, 23, 24, 25, 26, 27, 28, 29, 30, 31, 32, 33, 34]. Unlike miRNAs, which have multiple mRNA targets due to the imperfect base pairing, siRNAs can only recognize one specific mRNA to be fully complementary towards the binding region [21]. As a result, siRNAs are regarded as powerful genetic tools for therapeutic applications across different diseases. The approval of the first siRNA‐based drug, Patisiran, by the United States Food and Drug Administration (FDA) in 2018 marked the beginning of a new era in the utilization of RNAi machinery for the development of oligonucleotide therapeutics [6]. As of now, there are a total of six siRNA‐based drugs available on the market, targeting a spectrum of medical conditions including Dyslipidemia (Inclisiran) [35], Hereditary transthyretin amyloidosis with cardiomyopathy (Vutrisiran) [36, 37] or with polyneuropathy(Vutrisiran, Patisiran) [38], acute hepatic porphyria (AHP)(Givosiran) [39], and primary type 1 hyperoxaluria (PH1)(Lumasiran, Nedosiran) [40, 41]. Yet, despite the significant advancements of gene‐targeting therapies in other disease areas, clinical translation of siRNA therapeutics in CVDs remains slow, underscoring the necessity for developing efficient delivery systems for protecting nucleotides and synchronously achieving targeted delivery to specific sites beyond the liver [42].

In Section 3, it is discussed the current progress and challenges of siRNA delivery are discussed with a focus on MI and atherosclerosis. In Section 4, the latest nanoparticle‐based biological materials and emerging approaches are discussed for efficient cardiac‐oriented siRNA delivery. For miRNA‐based therapeutics in CVDs, readers may refer to more comprehensive reviews regarding the breakthroughs of the latest studies [25, 26, 27, 28, 29, 30, 31, 32, 33, 34, 35, 36, 37, 38, 39, 40, 41, 42, 43, 44, 45].

## 3 siRNA Therapeutics in Cardiovascular Diseases

Given the complexity and specialized nature of cells within the myocardium, siRNA‐based therapeutics hold great potential to anchor different targets based on clinical need, advancing the landscape of precision medicine. This section provides an overview of the latest achievements of siRNA therapeutics in CVDs, highlighting the key clinical opportunities in the rapidly emerging field of cardiac gene therapy.

### 3.1 Overview of CVDs

CVDs refer to a collective term designating the disorders of the heart and blood vessels, mainly including coronary heart disease, cerebrovascular disease, rheumatic heart disease, and other conditions [46, 47]. Irrespective of age and gender, the leading risk factors for CVDs are smoking, diabetes, hypertension, dyslipidemia, and physical inactivity [48]. Coronary heart disease (CHD) develops as a result of blockages within the blood vessels that supply oxygen to the heart muscle [49]. These blockages can partially or completely obstruct one or more coronary arteries, conditions normally referred to as ischemic heart disease and acute MI [49]. The major cause for CHD is atherosclerosis, a chronic inflammatory condition initiated by the accumulation of low‐density lipoprotein (LDL) cholesterol particles within the arterial walls [50]. The formation of atherosclerotic plaques with a prominent necrotic core, or a high susceptibility to rupture, can precipitate ischemic events such as MI [50, 51]. Currently, clinical guidelines for managing chronic and acute heart diseases focus on preventing or mitigating disease progression by targeting modifiable risk factors and restoring arterial blood flow through surgical procedures [4, 5, 6, 7, 8, 9, 10, 11, 12, 13, 14, 15, 16, 17, 18, 19, 20, 21, 22, 23, 24, 25, 26, 27, 28, 29, 30, 31, 32, 33, 34, 35, 36, 37, 38, 39, 40, 41, 42, 43, 44, 45, 46, 47, 48, 49, 50, 51, 52]. Yet, the global burden of CVD remains substantial, underscoring the need for continued investigation into novel therapeutics in this field.

### 3.2 Myocardial Infarction (MI)

**FIGURE 2:**
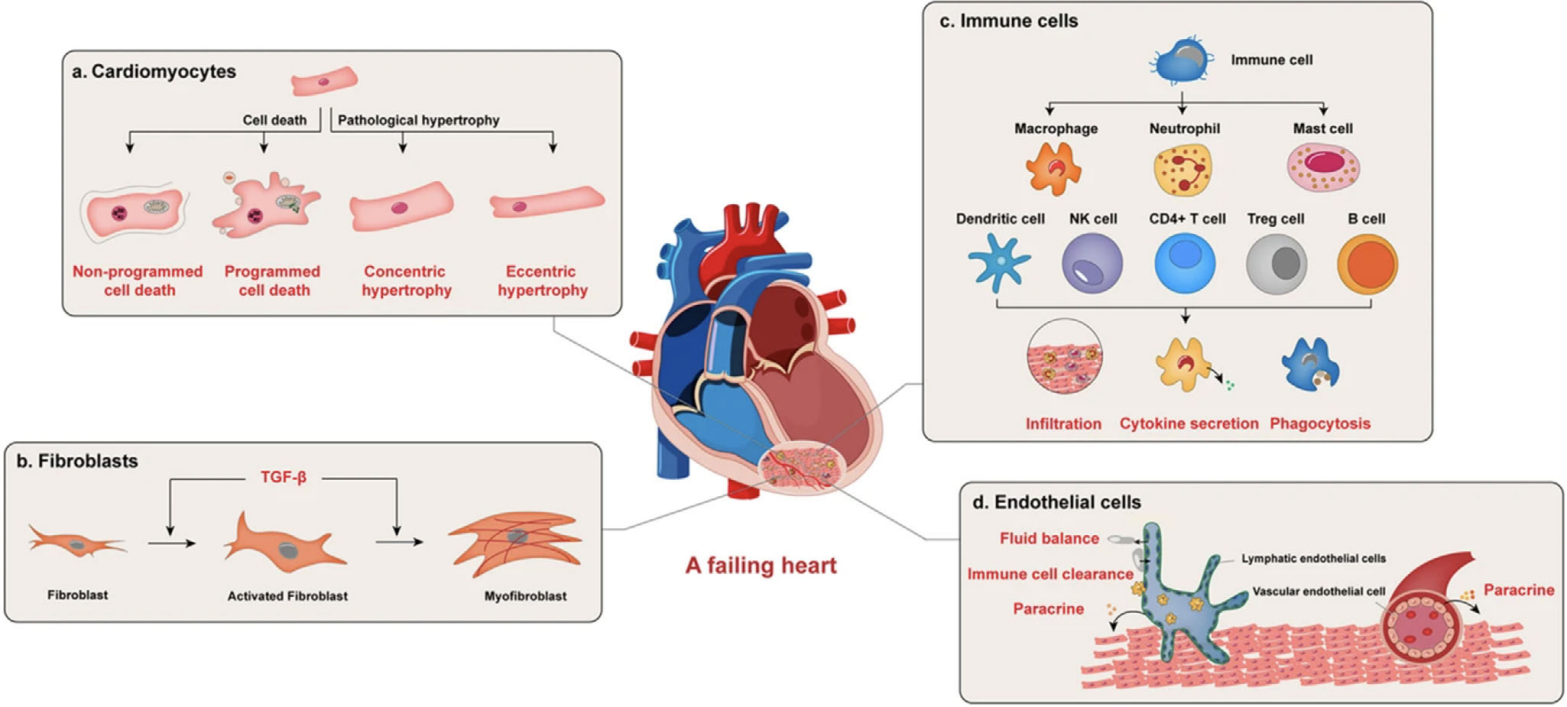
Functions of different cell types in a failing heart. Heart failure is a complex process that involves multiple cell types in the heart. Under stress, cardiomyocytes undergo either pathological hypertrophy or cell death. Hypertrophy led to cardiomyocyte dysfunction, while non‐programmed or programmed cell death led to cardiomyocyte loss. Cardiac fibrosis is another form of cardiac remodeling. It mainly involves fibroblast activation and conversion to myofibroblast. Various immune cells also contribute to heart failure. These cells infiltrate the injured myocardium, secrete cytokines, and clear unwanted material to regulate inflammation, regeneration, and function of other cell types in the failing heart. Both vascular endothelial cells (VECs) and lymphatic endothelial cells (LECs) regulate cardiac function. VECs affect neighboring cardiac cells through paracrine factors. LECs regulate cardiac regeneration after infarction by maintaining fluid balance, promoting immune cell clearance, and also secreting paracrine factors. Reproduced with permission [54]. Copyright 2022, Springer Nature.

MI, commonly referred to as a “heart attack”, occurs due to a significant reduction or complete cessation of blood flow to a segment of the myocardium [53]. For the human heart, the acute occlusion can result in liquefactive necrosis of myocardial tissue, whereas the heart itself lacks intrinsic capacity for self‐repair, leading to cardiac dysfunction and HF [11, 12, 13, 14, 15, 16, 17, 18, 19, 20, 21, 22, 23, 24, 25, 26, 27, 28, 29, 30, 31, 32, 33, 34, 35, 36, 37, 38, 39, 40, 41, 42, 43, 44, 45, 46, 47, 48, 49, 50, 51, 52, 53]. With achievements in molecular studies, distinct cell types were identified to be involved in maintaining cardiac function and regulating heart remodeling, providing potential therapeutic targets for precision medicine (Figure 2) [54, 55, 56].

To bring about cardiac regeneration, current preclinical research has focused on inducing cardiomyocytes (CMs) proliferation, generating new cardiac muscle cells via differentiation of precursors, reprogramming fibroblasts, and modulating inflammation to prevent adverse remodeling [11, 12, 13, 14, 15, 16, 17, 18, 19, 20, 21, 22, 23, 24, 25, 26, 27, 28, 29, 30, 31, 32, 33, 34, 35, 36, 37, 38, 39, 40, 41, 42, 43, 44, 45, 46, 47, 48, 49, 50, 51, 52, 53, 54, 55, 56, 57, 58, 59, 60, 61]. In a recent preclinical study, revascularization has been shown with an endothelium‐targeted nanosystem [62]. Inhibition of ICAM‐1 with siRNA nanomedicine improved cardiac systolic function, reduced pro‐inflammatory responses, and promoted angiogenesis via a combination of CXCL12 chemokine‐based therapy [62].

In addition to revascularization, heart regeneration has been achieved by interrupting the Hippo signaling pathway using siRNA therapeutics [63]. During pathological cardiac remodeling, Hippo signaling has major cell‐intrinsic roles in maintaining heart size and homeostasis, providing great potential for therapeutic regulation post‐MI [64, 65]. One example is provided by the Sav1 blockage using siRNA therapies [63]. Upon homing to the inflamed myocardium, sustained release of siSav1 rescued cardiac functions and reduced heart apoptosis in a rat myocardial ischemia reperfusion (IR) injury model [63]. Subsequent preclinical work in pig models further demonstrated efficacy and renewability [63]. Similar conclusions were reached by previous studies uncovering the potency of siRNA therapies towards the Hippo pathway [66, 67]. In general, cell signaling pathways have critical roles as a regulating network involving the pathophysiological conditions post‐heart ischemic injury. For example, inhibition of the Toll‐like receptor 4 (TLR4)/MyD88/nuclear factor‐κB (NF‐κB) and transforming growth factor‐beta (TGF‐β) signaling pathways mitigates excessive inflammatory responses and reduces cardiac fibrosis, whereas the phosphoinositide 3‐kinase (PI3K)/Akt and mitogen‐activated protein kinase (MAPK) pathways are more closely associated with the regulation of cell death programs [66, 67, 68, 69]. The importance of molecular targets involved in cell signaling events is undeniable, particularly in providing therapeutic potential by utilizing RNAi‐based genetic tools. Thus, a deeper understanding of the underlying mechanisms and explorations of pathways crosstalk may be beneficial for advancing novel siRNA therapeutics for MI or IR injury, from bench to bed eventually.

Another scenario of siRNA therapeutics in heart regenerative medicine is directing stem cell differentiation. Human embryonic stem cells (hESCs) and induced pluripotent stem cells (iPSCs) are promising cell sources for differentiating into CMs for regenerative purposes; yet, low transformation efficiency may result in mixtures of mature and immature cell populations [70, 71]. To address this, researchers attempt to downregulate Kinase Insert Domain Receptor (KDR) by lipid‐like materials‐mediated siRNA delivery system, which can prevent the differentiation of an entire germ layer and direct stem cell differentiation towards desired cell types [71]. Such an approach further integrates siRNA therapeutics into the realm of tissue repair and regeneration, demonstrating significant potential for advancing heart regeneration.

### 3.3 Atherosclerosis (AS)

**FIGURE 3:**
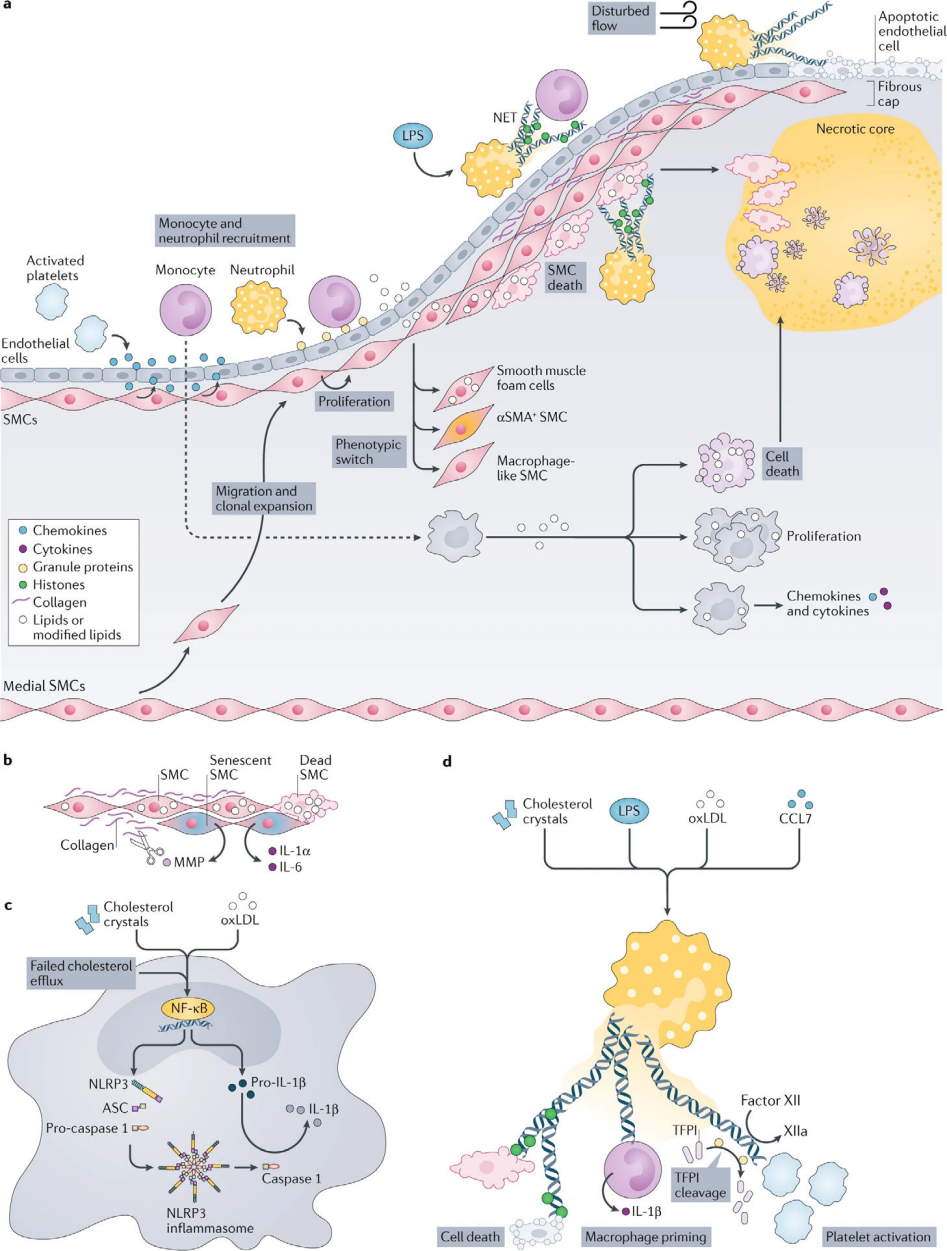
(a) Overview of inflammatory processes. At the early stages of atherosclerosis, activated platelets secrete chemokines (such as C‐C motif chemokine 5 (CCL5)) that promote adhesion of monocytes and neutrophils. Neutrophils themselves secrete chemotactic granule proteins (including cathelicidin, cathepsin G, and CCL2), thus paving the way for arterial monocyte infiltration. The chemokine milieu is supplemented by chemokines secreted by activated smooth muscle cells (SMCs), such as CCL2 and CCL5. In progressing atherosclerotic lesions, medial SMCs migrate towards the developing fibrous cap where they undergo clonal expansion. SMC lipid loading triggers phenotype switching towards SMCs that express α‐smooth muscle actin (αSMA+ SMCs), macrophage‐like SMCs, and smooth muscle foam cells. Heightened lipid loading of SMCs induces SMC apoptosis and, if not cleared quickly, necrosis. SMCs also undergo cell death after interaction with histone H4 presented in neutrophil extracellular traps (NETs). NET‐associated cytotoxicity is observed during plaque erosion when NETs released at sites of disturbed flow induce endothelial cell desquamation. In systemic infections with Gram‐negative organisms, which produce lipopolysaccharide (LPS), NET‐associated histones promote the adhesion of monocytes, hence contributing to accelerated plaque growth under these conditions. Monocyte‐derived macrophages ingest modified lipids and, in response, secrete inflammatory chemokines and cytokines. Excessive lipid uptake triggers macrophage proliferation or even cell death. (b–d) Core inflammatory processes fueled by SMCs (part b), macrophages (part c), and neutrophils (part d). (b) Cholesterol uptake induces cell death in SMCs. SMC death, in turn, reduces the amount of extracellular matrix that is produced, which further fuels SMC death. Senescent SMCs release pro‐inflammatory cytokines and matrix‐degrading enzymes, including matrix metalloproteinases (MMPs). (c) Priming and activation of the NACHT, LRR, and PYD domains‐containing protein 3 (NLRP3) inflammasome. Priming by cholesterol crystals, modified lipids such as oxidized low‐density lipoproteins (oxLDL) or impaired cholesterol efflux triggers the nuclear factor‐κB (NF‐κB) signalling pathway, promoting the transcription of NLRP3 and pro‐IL‐1β. Assembly of the NLRP3 inflammasome induces activation of caspase 1, which cleaves pro‐IL‐1β into mature IL‐1β. (d) Release of NETs is triggered by cholesterol crystals, LPS, modified lipids, and chemokines such as CCL7. NETs exert cytotoxicity by means of NET‐resident histones, prime the NLRP3 inflammasome in macrophages, and induce coagulation by cleavage of factor XII and tissue factor pathway inhibitor (TFPI), and by direct platelet activation. Reproduced with permission [73]. Copyright 2021, Springer Nature.

As the major cause of acute myocardial ischemia, atherosclerosis is a chronic inflammatory disorder driven by lipid accumulation in the arterial wall. With disease progression, unstable plaques with necrotic cores can rupture and induce the formation of occlusive thrombi, eventually leading to clinical manifestations such as MI [72]. Along with the chronic inflammation, there are three major cell types contributing to the induction of the arterial lesions, including smooth muscle cells (SMCs), macrophages, and neutrophils. The underlying molecular cross‐talks within different inflammatory pathways were extensively discussed by Peter et al. (Figure 3) [73]. Considering the development of atherosclerosis extends for long periods of time along with the inflammation cascades, there exists a “window of opportunity” for implementing treatments to reduce the disease burden. A unique aspect of atherosclerosis is the crucial role of low‐density lipoprotein (LDL) particles in the introduction of atherosclerosis [50]. Depending on this, the current therapeutic strategies mainly focus on regulating the levels of LDL within blood vessels [74]. The first siRNA‐based therapeutics for anti‐atherosclerosis came out in December 2021, as an inhibitor for targeting PCSK9 mRNA, demonstrating efficacy of cholesterol lowering and disease risk reduction [75]. This achievement signifies a breakthrough in gene therapy for anti‐atherosclerotic interventions. However, the latest follow‐up studies from ORION‐3 have reported side effects associated with Inclisiran treatments, including nasopharyngitis and adverse events at the injection site, underscoring the need for further efforts to enhance efficacy and safeguard biosafety for long‐term application [76].

One significant advantage of RNAi‐based therapeutics over conventional therapies is to affect targets that cannot be modulated with conventional pharmaceuticals, allowing for precise intervention towards specific cell subsets [77]. This unique aspect confers them as robust genetic tools for tuning immunity in the pathological progression of atherosclerosis. Over the past five years, researchers have devoted intensive efforts to modulating proatherogenic macrophages as a strategy to combat lipid‐hardening vascular disease [50]. In one study, a lesional macrophage‐targeted nanosystem successfully achieved gene silencing by intravenous delivery of siRNA counteracting CaMKIIγ (Ca^2+^/calmodulin‐dependent protein kinase γ), a modulator involved in plaque vulnerability [78]. Downregulation of CaMKIIγ promoted efferocytosis, increased fibrous cap thickness, and inhibited atherosclerotic plaque necrosis, uncovering the feasibility of siRNA therapeutics in atherosclerotic macrophages [78]. Further promise needs to be proved in larger animal models prior to clinical translation. Similarly, subsequent studies have been reported to resolve pro‐inflammatory cascades within the atherosclerosis microenvironment, to better stabilize plaque and support efferocytosis function [79, 80, 81].

Foam cells, a hallmark of atherosclerosis, are commonly derived from macrophages, dendritic cells, and vascular smooth muscle cells (VSMCs) [50, 51, 52, 53, 54, 55, 56, 57, 58, 59, 60, 61, 62, 63, 64, 65, 66, 67, 68, 69, 70, 71, 72, 73, 74, 75, 76, 77, 78, 79, 80, 81, 82]. In addition to VSMCs‐derived foamy cells, which occupied approximately 50% of all foam cells within human lesions, macrophages contribute to a considerable number of lipid‐rich foamy cells in atherosclerotic plaques [83, 84] With the increased internalization of oxidized low‐density lipoprotein (oxLDL) and accumulation of lipid droplets, macrophage foamy cells gradually occur, leading to fatty streaks and the formation of plaque lesions [85]. As the disease progresses, lipid‐laden foamy macrophages experience a loss of efferocytosis function, leading to inefficient lipid clearance and disrupted lipid metabolism [86]. This imbalance eventually induces the formation of a necrotic core, which is associated with unstable rupture‐prone lesions [86]. Moreover, previous studies identified a subset of foamy triggering receptor expressed on myeloid cells 2 (TREM2)‐expressing macrophages, a unique population that is not inflammatory, whereas it shows impaired cholesterol efflux capacity and cellular catabolic processes, serving as a therapeutic target for regulating lipid homeostasis [87, 88]. Recently, targeting foamy macrophages represents novel therapeutic strategies for stabilizing plaques and suppressing the progression of atherosclerosis [83]. In one study, the incorporation of hyaluronic acid (HA) provided substantial activity for nanotherapeutics to target foamy macrophages, leading to efficient delivery of siRNA silencing LOX‐1, reducing lipid accumulation level and lesional areas in small animal models [89]. In subsequent work, this siLOX‐1 therapeutics was further combined with statin drug atorvastatin calcium (AT) for promoting efflux of lipids in both macrophages and endothelial cells, achieving a synergistic treatment efficacy to regress atherosclerotic plaques [79].

Overall, the abovementioned preclinical studies highlight the pivotal role of foamy macrophages involved in the pathogenesis of atherosclerosis. Future endeavors should focus on the identification of distinct stages of foam cells and the potential for homeostatic modulation by siRNA nanomedicine.

## 4 Nanomedicines for Cardiovascular Diseases

**FIGURE 4:**
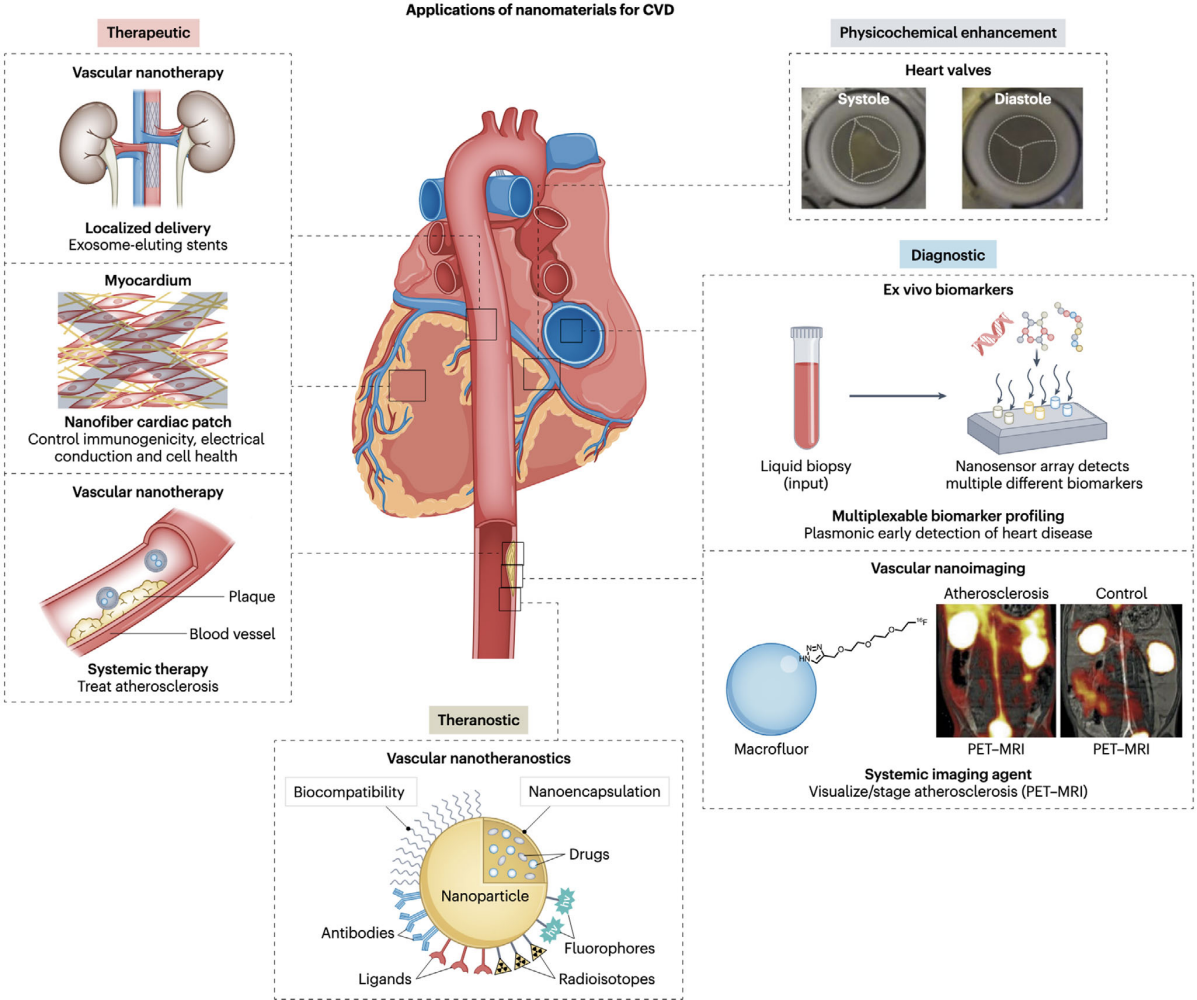
Cardiovascular nanomedicine applications can be divided into four major application classes, spanning new therapeutics and diagnostics to theranostics and nanomaterials for physicochemical enhancements. Therapeutics and diagnostic imaging include both systemic and localized nanodelivery applications. Diagnostics may also involve ex vivo applications in which nanomaterials amplify signal by leveraging electrical, magnetic, acoustic, and optical signal reporting and/or boosting capabilities, for example, to drive faster, more efficient, or more highly parallelized screening and response‐to‐therapy assessment strategies. Theranostics integrates therapeutic and diagnostic modalities, typically into a single nanomaterial. Physicochemical enhancements encompass the application of nanomaterials to boost and/or beneficially modulate a bulk material's electrical, structural, chemical, immunological, and/or mechanical properties, often to support extended contact with in vivo biology. Reproduced with permission [9]. Copyright 2023, Springer Nature.

Over the past decades, advances in understanding the mechanisms underlying the pathogenesis of CVDs have set the stage for significant breakthroughs in the medical field [9]. Nanomedicines represent a promising frontier in the treatment and management of cardiovascular diseases, offering the potential for more effective, precise, and personalized therapies [90, 91]. The leading role of nanotechnology in biomedical applications is primarily driven by its flexible controllability in engineering physicochemical properties [9, 10, 11, 12, 13, 14, 15, 16, 17, 18, 19, 20, 21, 22, 23, 24, 25, 26, 27, 28, 29, 30, 31, 32, 33, 34, 35, 36, 37, 38, 39, 40, 41, 42, 43, 44, 45, 46, 47, 48, 49, 50, 51, 52, 53, 54, 55, 56, 57, 58, 59, 60, 61, 62, 63, 64, 65, 66, 67, 68, 69, 70, 71, 72, 73, 74, 75, 76, 77, 78, 79, 80, 81, 82, 83, 84, 85, 86, 87, 88, 89, 90, 91, 92]. In the context of CVDs, the applications of nanotechnology are mainly involved in therapeutics and diagnostics, encompassing a wide range of conditions affecting the heart and blood vessels [9, 10, 11, 12, 13, 14, 15, 16, 17, 18, 19, 20, 21, 22, 23, 24, 25, 26, 27, 28, 29, 30, 31, 32, 33, 34, 35, 36, 37, 38, 39, 40, 41, 42, 43, 44, 45, 46, 47, 48, 49, 50, 51, 52, 53, 54, 55, 56, 57, 58, 59, 60, 61, 62, 63, 64, 65, 66, 67, 68, 69, 70, 71, 72, 73, 74, 75, 76, 77, 78, 79, 80, 81, 82, 83, 84, 85, 86, 87, 88, 89, 90, 91, 92, 93, 94]. The schematic illustration indicating the breakdown of therapeutic and diagnostic cardiovascular nanomedicine applications is shown below in Figure 4. This section outlines the fundamental design considerations for a gene delivery system based on current mechanistic insights. Furthermore, recent progress in commonly utilized nanosystems for cardiovascular gene therapy is categorized and detailed. Finally, advanced vehicles that have reached clinical trials have been highlighted, followed by discussions on current limitations that require further optimization for the bench‐to‐bedside transition.

### 4.1 General Requirements for the Delivery Systems

**FIGURE 5:**
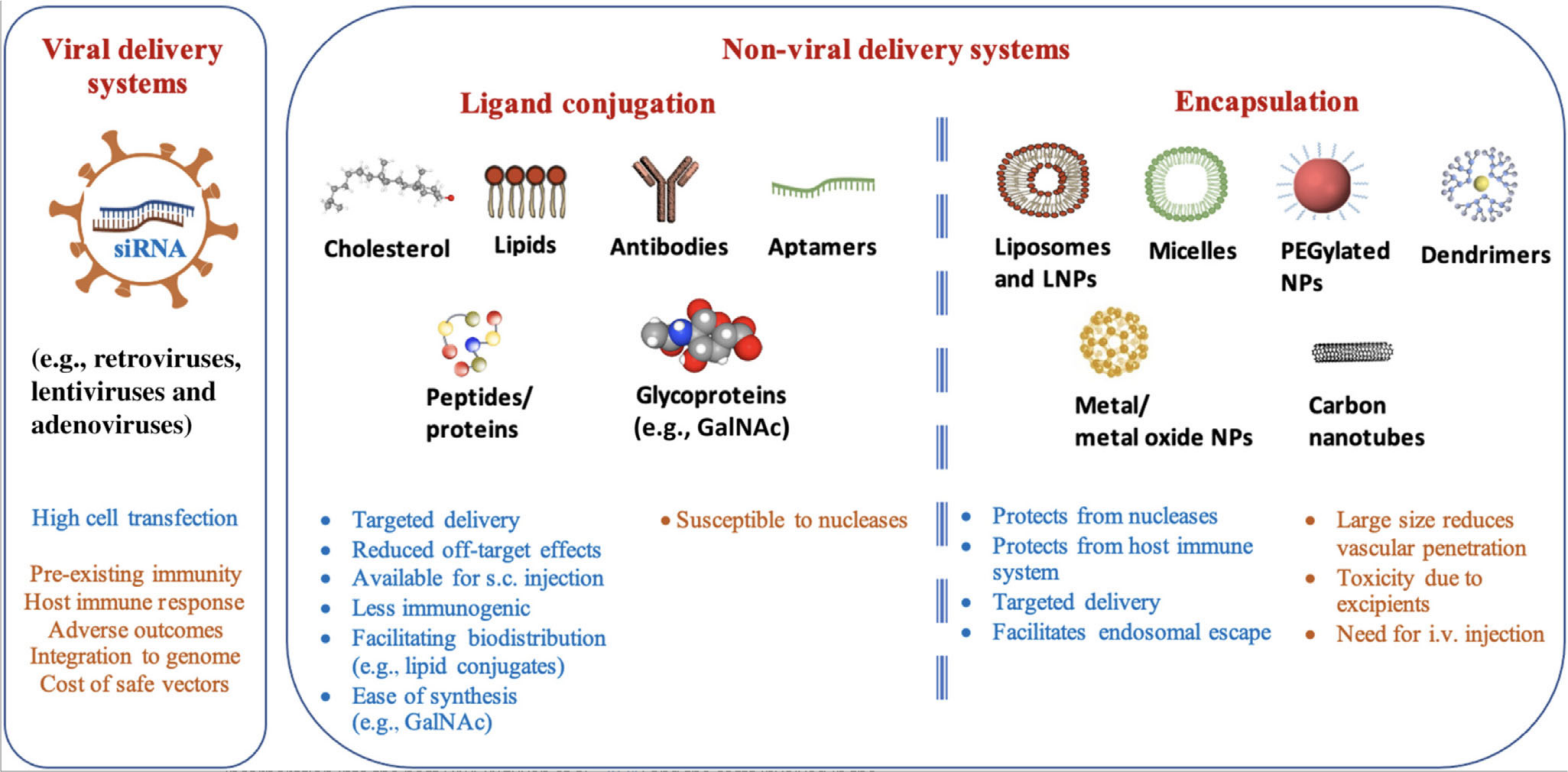
Small interfering RNA (siRNA) delivery strategies (blue text: advantages; orange text: disadvantages). GalNAc, N‐acetylgalactosamine; LNPs, lipid‐based nanoparticles; NPs, nanoparticles; PEG, polyethylene glycol. Reproduced with permission [97]. Copyright 2022, WILEY‐VCH Verlag GmbH & Co. KGaA, Weinheim.

RNA interference machinery has shown great promise in the precise targeting of pathological molecules, whereas naked siRNAs possess several limitations, including instability, poor pharmacokinetic behavior, and potential off‐target effects, underscoring the necessity to develop an efficient delivery system [95]. Currently, extensive preclinical investigations and clinical studies have focused on chemical modifications (e.g., GalNAc–siRNA conjugates) and delivery platforms (e.g., lipid nanoparticles (LNPs)), and the latter can be divided into viral and non‐viral delivery systems (Figure 5) [96, 97].

The efficiency of modified siRNA bioconjugates is highly dependent on the modification site on the backbone. For example, the replacement of the 2’OH group with 2’F and 2’O‐Me on double strands of siRNAs showed increased duration for gene silencing [98]. In contrast, the bulkier 2’O‐Me modification may reduce the activity of RNA interference, indicating the importance of identifying an optimal modification pattern to concomitantly achieve RNA protection and silencing efficacy [98]. Furthermore, it has been demonstrated that the patterns for conjugation were closely associated with the in vivo behavior of siRNA therapeutics, such as tissue‐specific accumulation and long‐term stability [98, 99]. One of the systematic works was conducted by Matthew et al., where they compared partially or fully chemically modified siRNAs on the efficacy of in vivo delivery [99]. An asymmetric, cholesterol‐modified siRNAs were adopted as a model; they further labelled the siRNA with fluorescein Cyanine3 (Cy3) for visualizing biodistribution after intravenous (i.v.) or subcutaneous (s.c.) administration at a dosage of 10 mg/kg [99]. Authors found that injection with fully modified siRNAs resulted in robust accumulation in major organs, including liver, kidney, spleen, fat, and skin, whereas only marginal fluorescence was observed in liver and kidney in the group injected with partially modified siRNAs [99]. This study, combined with other findings, collectively underscores the significance of rational design of conjugation schemes for efficient siRNA delivery in vivo [99, 100, 101]. For more comprehensive details on the implications of chemical modifications for siRNA activity, readers may refer to other reviews focused on bioconjugate‐mediated siRNA delivery [102, 103, 104].

Apart from chemical modifications, there has been a robust effort in developing nanoparticles (NPs)‐based siRNA delivery vehicles. Unlike viral vectors, which carry concerns of immunogenicity and cytotoxicity, non‐viral‐based NPs have drawn emerging attention due to their advantages in tunable properties, biodegradability, and economic feasibility [95, 96, 97, 98, 99, 100, 101, 102, 103, 104, 105]. Yet, to secure efficient siRNA therapeutics towards the intended sites, several requirements need to be considered for advancing the drug delivery system. Meanwhile, understanding the underlying mechanisms throughout the siRNA transportation process is the prerequisite for resolving obstacles and achieve therapeutics outcomes. In general, from a pharmaceutical point of view, design requirements can be categorized into three aspects: (1) protect siRNA payloads from degradation; (2) tailored delivery towards specific cells or tissues; and (3) efficient release of siRNA into the cytoplasm to fulfill RNAi machinery.

Solid encapsulation of RNA molecules is the foremost step for optimizing the fabrication delivery systems, which depend on the physicochemical properties of chosen materials and binding affinity between nucleic acids and biomaterials [95]. For instance, cationic polysaccharides (e.g., chitosan, cyclodextrin) can condense anionic siRNA within the polymer matrix via electrostatic interactions [102]. Notably, the complexation efficiency can vary depending on the polymer MW. It has been reported that higher MW chitosan contributes to efficient transgene expression and increased stability, whereas lower MW chitosan may facilitate intracellular release [106]. Besides physicochemical properties, the synthesis conditions, such as buffer solutions and pH value, can also influence the RNA encapsulation, which has been elaborated elsewhere [95]. In fact, the most crucial parameter for electrostatic interaction is the N/P ratio, which refers to the ratio of positively charged amino groups (N = nitrogen) to negatively charged nucleic acid phosphate groups (P = phosphate) [107]. High N/P ratios may enhance the complexation degree of polymer/nucleic acid complex, whereas it can concomitantly carry concerns of cytotoxicity due to the excess cationic charge [107]. Hence, it is crucial to determine proper N/P ratios for condensing siRNAs without compromising biological effects. As Borja et al. reported, variations in N/P ratios (5, 20, 60, 120) between chitosan and siRNA influenced the stability and biological interactions of resultant NPs [108]. The organ distribution pattern was revealed at 1 h after oral gavage; higher levels of siRNA accumulation in major organs were observed from the N/P 120 group, suggesting the improved NPs’ stability may facilitate the precise delivery of siRNA with marginal pre‐mature degradation [108]. The optimized N/P ratio can also be affected by chemical modifications on the backbone of the polysaccharide. For example, in a different study performed by Fen et al., the developed NPs fabricated by polysaccharide derivative showed an optimal N/P ratio at 20, reaching the highest transfection value at 98.7% ± 3.1% [109].

Besides the ionic bindings that are mainly used in the fabrication of polysaccharide−siRNA nanocomplexes, the hydrophobic interaction has also been reported as the general methodology for RNA condensation [95, 96, 97, 98, 99, 100, 101, 102, 103, 104, 105, 106, 107, 108, 109, 110]. Hyaluronic acid (HA) is a natural biomacromolecule that is commonly used for coating or assembling with cationic polymers for siRNA nanocarrier fabrication [111]. Recently, Maruthibabu et al. reported for the first time on an anionic, non‐viral siRNA delivery method [112]. Significant thermal stabilization of siRNA duplex was observed with all sizes of HA, where maximized in the presence of 200 kDa HA (ΔTm = 1.48°C) [112]. The binding mechanisms behind siRNA‐HA were investigated via molecular dynamics (MD) simulation, by which they discovered favorable van der Waals interactions and hydrogen bonds that accounted for RNA stabilization [112].

### 4.2 Establishing the Delivery Framework

**FIGURE 6:**
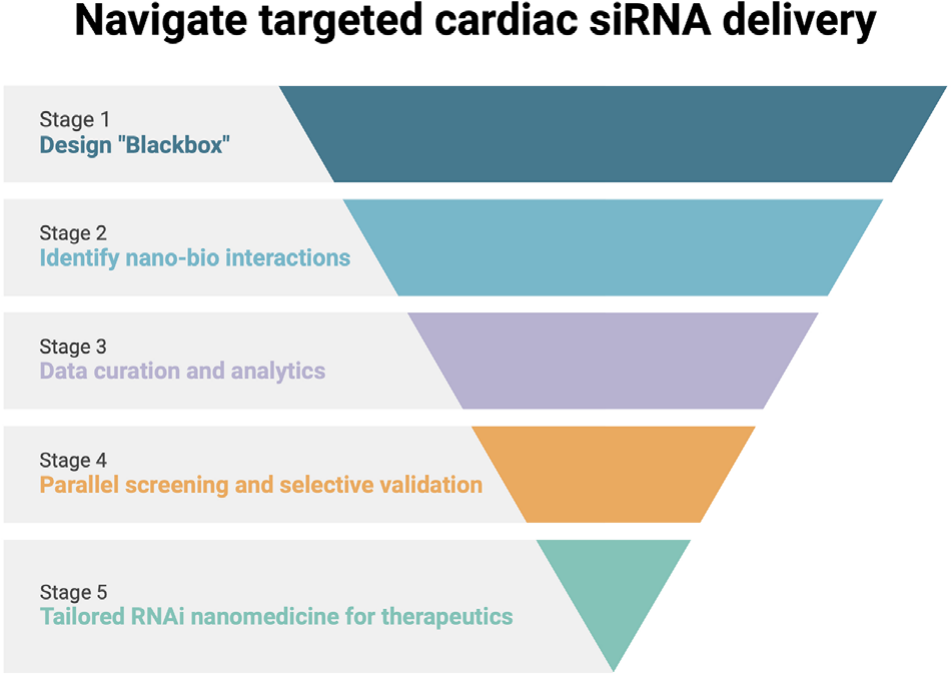
The schematic framework for advancing targeted cardiac siRNA delivery. The figure was created with BioRender.com.

Efficient delivery of genetic biologics to specific target sites within the body is a critical prerequisite for achieving successful diagnostics and therapeutic outcomes. This process necessitates a thorough consideration of various factors throughout the nanoparticle's biological journey. From this perspective, a rational design framework is crucial for directing therapeutic agents to the target site, while minimizing off‐target effects and reducing potential immunogenicity to the greatest extent (Figure 6). In this subsection, we provide a concise overview of the nanoparticle journey following administration and propose strategies for navigating and optimizing delivery systems to advance the paradigm of precision RNAi nanomedicine.

#### 4.2.1 Identify Bio‐Nano Interactions

**FIGURE 7:**
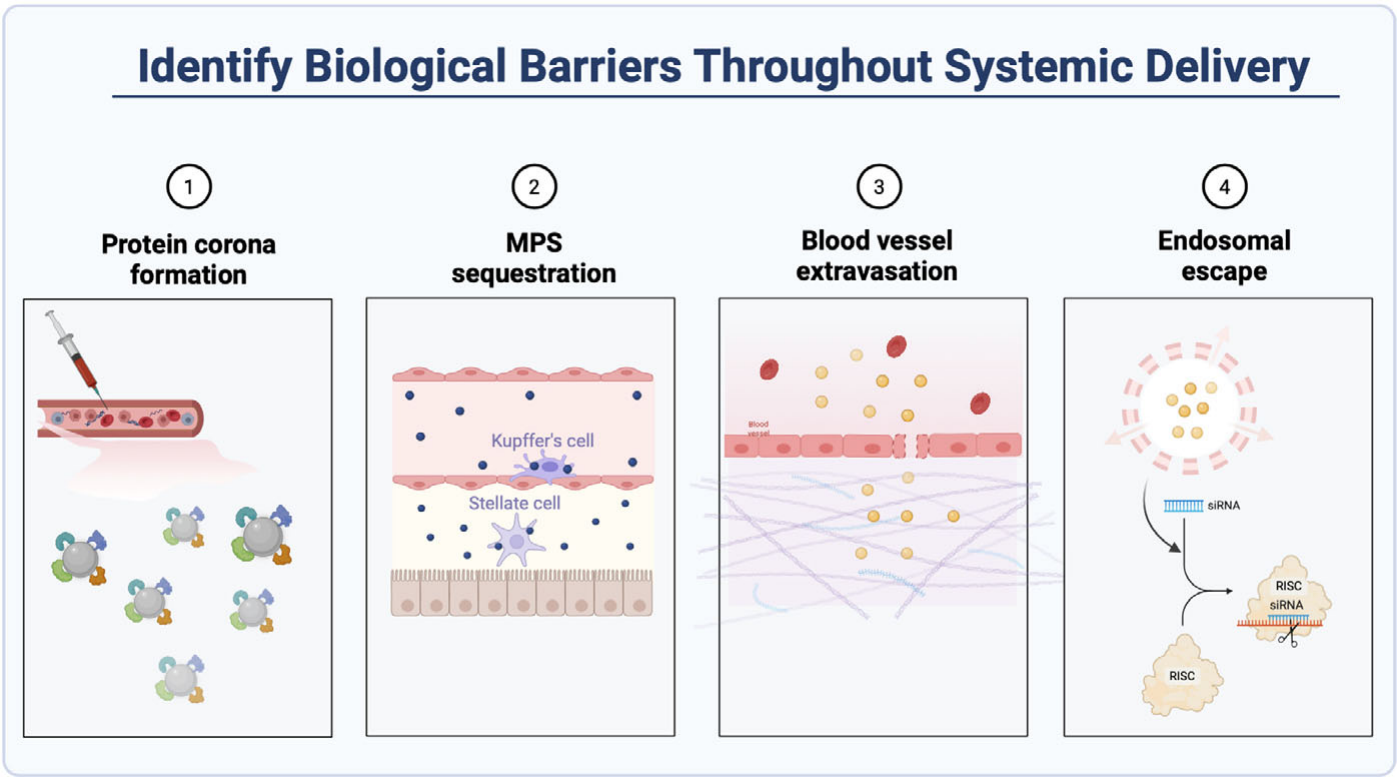
An overview of biological barriers throughout nanoparticles’ systemic delivery. The figure was created with BioRender.com.

The delivery efficacy of nanoparticles is modulated by numerous factors, encompassing their chemical compositions, physicochemical characteristics, and interactions within biological environments [113]. The design criteria should, in principle, prioritize stability to protect payloads while maintaining simplicity to facilitate clinical translation [114]. In the context of RNAi delivery systems, it is important to consider several requirements with respect to safety, targetability, and efficacy, which were discussed in Section 4.1. Beyond that, identifying the bio‐nano interactions alongside the administration route may contribute to reducing the potential barriers and promote direct delivery to the lesional site (Figure 7).

Upon intravenous administration, the major obstacle towards nanoparticles’ delivery is the sequestration by the mononuclear phagocyte system (MPS), resulting from tissue macrophages (mainly liver and spleen), blood monocytes, and bone marrow precursors [115]. The undesired non‐specific interaction, involving the adsorption of plasma proteins, begins with the opsonization of nanoparticles [116]. The formation of protein corona, in turn, may unpredictably alter surface properties, function, and avidity effects of nanoparticles, resulting in unfavorable challenges such as aggregation, rapid clearance, and off‐target effects [117]. Given that such conformational changes on nanomaterials depend on several factors, studies in the past decade investigated particles with different sizes, compositions, and surface chemistries that adsorbed biological corona [118, 119, 120, 121, 122, 123]. Considering these interfaces are dynamic and selective, future investigations are highly recommended to preclinically quantify NPs’ delivery and distribution in a specific environment.

Following protein absorption, the reconstructed patterns on the surface of nanomaterials can induce different NPs’ outcomes, such as being easily recognized by MPS [116] or triggering distinct cellular uptake mechanisms [124]. Accordingly, characterizing the protein corona composition on nanomaterials may offer a means to strategically influence their fate during cellular internalization and trafficking. For instance, Caracciolo and co‐authors investigated the biological activity of cationic liposomes correlated with protein compositions, where they found that NPs’ sequestration by circulating leukocytes was dominantly influenced by the surrounding proteins (such as immunoglobulins) [125]. In contrast, pre‐coated liposomes at high protein concentration (HP  =  50%) manifested prolonged blood circulation and enhanced uptake due to the poor enrichment of typical opsonin, offering a new strategy to modulate the behavior of liposomes via harnessing protein corona [125]. In addition, engineering strategies are developed that allow NPs to be stealth within the bloodstream, such as PEG coating and platelet membrane cloaking, avoiding recognition and clearance by physical barriers [126, 127].

Reaching targeted tissues/organs is the prerequisite for nanotherapeutics, contingent upon the nanoparticles' ability to extravasate and penetrate vascular structures [113]. Extravasation is influenced by various nanoparticle properties, including size, shape, surface chemistry, and the composition of the protein corona formed upon interaction with biological fluids [116, 117, 118, 119, 120, 121, 122, 123, 124, 125, 126, 127, 128]. Moreover, the heterogeneity of vascular permeability across different tissues and pathological states, particularly the transiently increased microvascular permeability in ischemic or infarcted myocardium, significantly impacts nanoparticle accumulation at target sites [129]. Thus, future efforts should prioritize engineering precision nanomedicines with active targeting or responsive capabilities to bypass these biological barriers and ensure selective cardiac repair. Additionally, integrating advanced imaging and computational modeling techniques may allow for real‐time monitoring and predictive analysis of nanoparticle extravasation patterns, enabling more precise targeting strategies.

#### 4.2.2 Endocytosis and Endosome Escape

The major advantages of non‐viral gene delivery vehicles lie in the potential to protect delicate therapeutics from degradation and target specific cells, followed by sustained release of drugs [130]. Yet, along with the intracellular uptake of NPs, different internalization pathways will be presented that can influence the delivery outcomes ultimately [130]. In general, the principal route for non‐viral vectors to enter cells is via endocytosis, which encompasses various pathways that are dependent on the physicochemical properties of the engineered nanoparticles [131]. More comprehensive reviews on distinct endocytic pathways have been highlighted by Parton et al. [130].

Upon internalization, evidence indicated that all major endocytic trafficking pathways converge at a “sorting station”, referred to as the early endosome, which functions to direct cargos into different intracellular destinations, such as recycled back to the cell surface or converted to a later‐endosomal counterpart [132]. The fate of nanocarriers sequestered within early endosomes can significantly influence the release of cargos into the cytoplasm. This process is often impeded by a physicochemical barrier known as endosomal escape [133]. Efficient endosomal escape is the prerequisite for successful release of siRNA payloads into the cytoplasm, where the RISC module is assembled and functions as a robust genetic tool [12]. To enhance endosomal escape, elucidating the mechanisms underlying the cytosolic release of siRNAs from endosomes may offer valuable insights for developing rational design strategies. To address this, efforts have been made to visualize the intracellular trafficking of siRNAs. For example, Zerial's lab has investigated the cytosolic release of siRNA via conjugating to the colloidal gold particles [134]. Their findings indicated the low efficiency (1%–2%) of escape from endosomes mediated by lipid nanoparticles (LNPs), which occurred within a limited “window of time” [134]. Moreover, this specific compartment of escape exhibits characteristics typical of both early and late endosomes [134].

**FIGURE 8:**
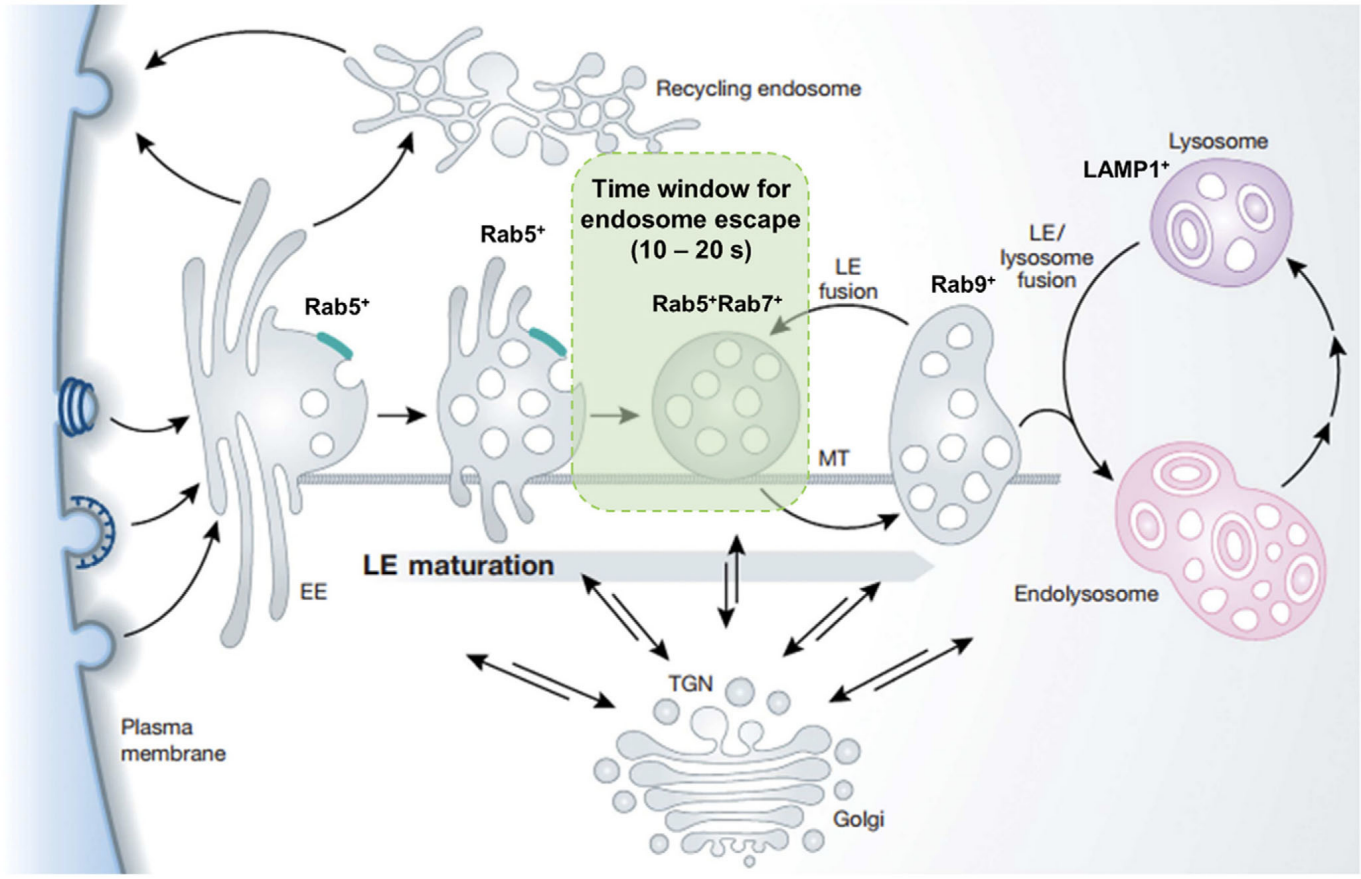
Endosome maturation process and the corresponding time window for siRNA endosome escape. After the primary endocytic vesicles form, the NPs containing vesicles sequentially form into early endosomes (EE) featuring Rab5^+^. EEs are moving in perinuclear space along microtubules (MT), where the conversion of Rab5^+^ EE to Rab7^+^ EE takes place. The endosome escape of siRNA will be initiated just before nascent late endosome (LE, Rab9^+^) undergo homotypic fusion reaction with Rab5^+^Rab7^+^ EE, where the majority of siRNA will be released within 10–20 s. The unreleased siRNA will accumulate in the LE and lysosome (LAMP1^+^) for degradation. Reproduced with permission [95, 136]. Copyright 2011, John Wiley & Sons, and Copyright 2021, Elsevier B.V., respectively.

Building on this, to better understand the cytosolic release of siRNAs and link it to knockdown mechanisms, an imaging approach similar to the high‐dynamic‐range (HDR) technique has been developed by Lieberman et al. [135]. Alexa Fluor 647–labeled siRNAs (siRNA‐AF647) were incorporated in LNPs and incubated with Hela cells. Fluorescence intensity of siRNA‐AF647 was captured primarily with two different exposure settings; with this method, the siRNA payloads have been detected to diffuse and fill into the cytosol within 10–20 s (Figure 8) [135, 136]. Based on this, they further explored the duration of release that occurred in a particular stage of endosomal maturation. Release occurred approximately 5.5 min after maximal GFP‐EEA1 intensity and 3 min after maximal GFP‐Rab5 intensity, indicating that the majority of release events occurred within 5–10 min after NPs endocytosis [135]. Moreover, following burst release, the authors also suggested that the targeted GFP fluorescence began to decrease ∼60 min after release. Along with previous studies, it was found that <2000 cytosolic siRNAs/cell are needed for inducing maximal knockdown [135]. Overall, these results suggested that the narrow yet efficient “window of opportunity” during the endosomal maturation process, which shares both early and late endosomal characteristics, plays an important role in controlling the siRNA release (Figure 8) [134, 135, 136, 137]. This provides a foundation for further investigation into the mechanisms of endosomal escape in NPs‐mediated RNAi therapeutics.

While the general focus has been given to LNPs‐based nanocarriers, the mechanisms of polymer‐based NPs for endosomal escape remain inconclusive, probably due to the diversity of materials and structural tunability. Different from the lipoplexes, subtle changes on the backbone of polymers may result in distinct endosomal escape mechanisms, such as membrane rupture and destabilization [95, 96, 97, 98, 99, 100, 101, 102, 103, 104, 105, 106, 107, 108, 109, 110, 111, 112, 113, 114, 115, 116, 117, 118, 119, 120, 121, 122, 123, 124, 125, 126, 127, 128, 129, 130, 131, 132, 133, 134, 135, 136, 137, 138]. Further attempts are needed to clarify the specific uptake of polymer‐based NPs and the corresponding siRNA release, advancing the knowledge on the underlying mechanisms behind the release kinetics.

### 4.3 Adeno‐Associated Virus (AAV)‐Mediated siRNA Therapy

Among the viral vehicles, adeno‐associated virus (AAV)‐based systems represent one of the most extensively investigated platforms for siRNA delivery in cardiovascular diseases, driven by their high transduction efficiency, favorable in vivo stability, and long‐term gene silencing effects [139, 140]. From a biological standpoint, AAV vectors are derived from non‐pathogenic parvoviruses with episomal persistence, enabling sustained genetic modulation in post‐mitotic tissues, such as the heart [141]. In addition, distinct serotypes and engineered capsid variants allow for differential tissue tropism, benefiting tailored siRNA delivery towards specific cell populations [142]. For instance, taking advantage of the superior affinity of adeno‐associated virus type 9 (AAV9) to myocardium, Su et al. utilized AAV9 to incorporate siRNA targeting the suppressor of cytokine signaling 3 (SOCS3), following in vivo administration, alleviating myocardial fibrosis and improving heart function were observed, demonstrating a promising approach for the treatment of diastolic heart failure [141]. Beyond the application of AAV9, other serotypes such as AAV6 and AAV8 have also demonstrated cardiac tropism in preclinical disease models [143, 144].

Despite the steady progress and inherent characteristics of AAV‐based delivery platforms, the path to clinical translation has been hindered. One major limitation refers to the preexisting adaptive immunity due to the presence of neutralizing antibodies (Nabs), which can compromise gene transfer efficacy [145]. Furthermore, this immunological barrier precludes the possibility of repeated dosing due to the adaptive immune recognition, leading to limited therapeutic effects following systemic administration [145, 146]. To circumvent this challenge, efforts have been made to counteract immunorecognition of AAV vectors, including enzymatic degradation, capsid engineering, pharmacological inhibition, and a biomimetic shield using exosomes/cell membranes [147, 148, 149]. Recently, Maiseyeu et al. reported a transformative approach using cargo‐free PLGA enhancer polymer (ePL). By modulating cardiac glucose uptake and glycosylation, the ePL system provides a modality‐independent strategy to enhance AAV heart tropism, delay blood clearance, and evade neutralizing antibodies, effectively addressing the critical safety‐efficacy bottleneck in cardiac gene therapy [150]. Looking forward, future research is encouraged to refine AAV‐based delivery systems with minimized immunogenicity and flexible dosing capacity.

### 4.4 Non‐Viral siRNA Delivery Systems

As concerns about the effectiveness of viral gene therapies persisted due to limitations like pre‐existing immunity and payload size constraints, scientists have fueled the search for alternative non‐viral drug delivery systems. In this subsection, we detail the expanding number of preclinical studies reported to utilize non‐viral nanoparticles for gene therapy in cardiovascular applications, including LNPs, polymeric NPs, exosomes, and polysaccharides. Finally, we introduced the clinical status of trials regarding genetic therapeutics for CVD. We also discussed the existing limitations and potential opportunities for future endeavors.

#### 4.4.1 LNPs and Polymeric NPs

**FIGURE 9:**
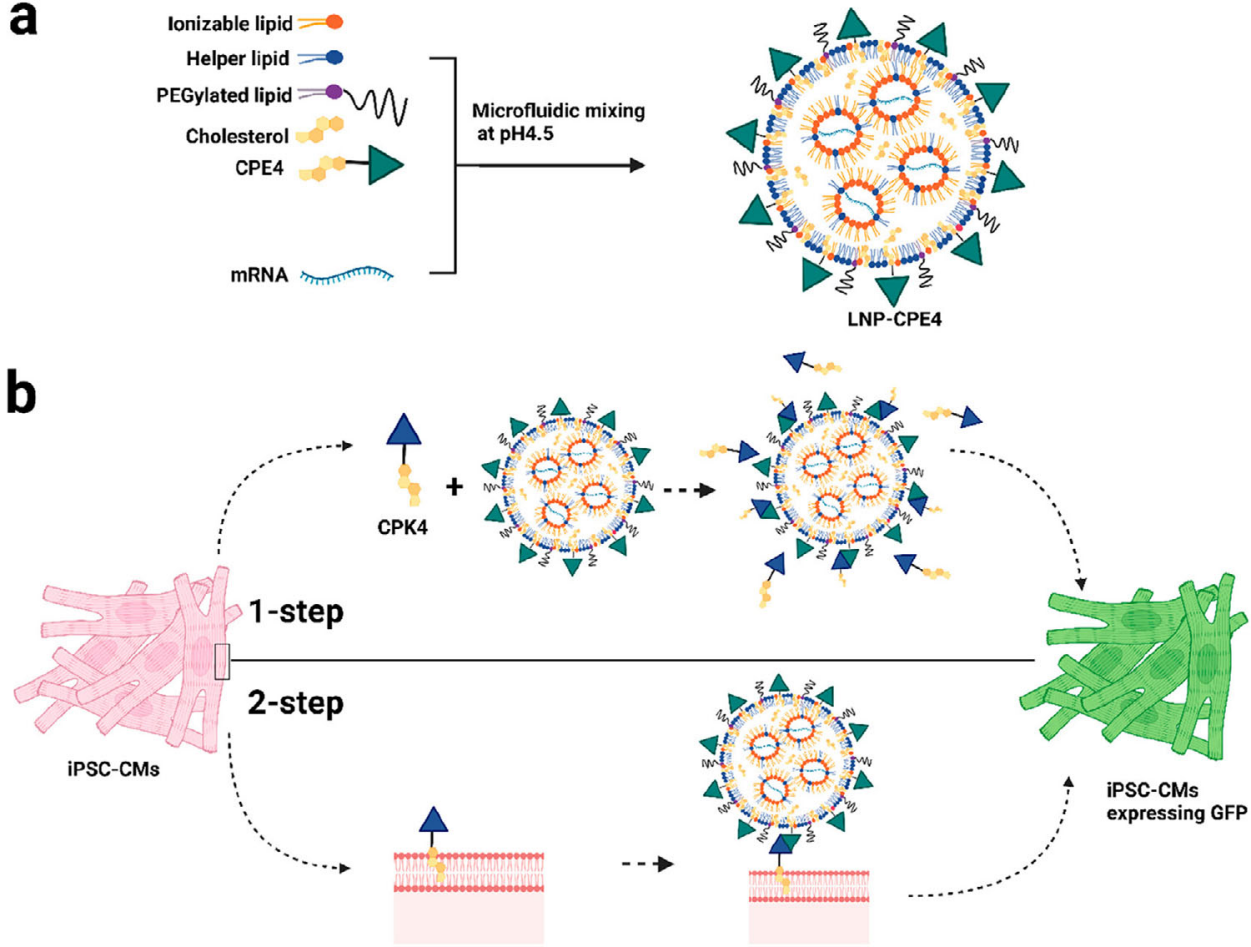
Schematic illustration of fusogenic LNP‐mRNA formulation. (a) Schematic Illustration of mRNA Encapsulating LNP‐CPE4. (b) Fusogenic Coiled‐Coil Peptide Modified Lipid Nanoparticles (LNPs) for EGFP‐mRNA delivery in iPSC‐CMs. Reproduced with permission [154]. Copyright 2023, American Chemical Society.

Among different nanomaterials, lipid nanoparticles (LNPs) have emerged as a powerful tool for gene therapy in the treatment of CVDs. These nanoscale carriers are designed to protect genetic drugs from multiple biological barriers, such as rapid clearance and off‐target effects. Recent advancements in LNP technology have further improved their biocompatibility and organ‐specific tropism, minimizing potential immune responses and off‐target effects [151, 152]. Variations in the composition of LNPs are closely associated with their biodistribution, cellular uptake, and delivery efficacy. In a recent study, the authors tested different LNP formulations with variations in their lipid composition [153]. By varying the type of helper lipid and the molar percentage of C12‐200 lipid, followed by encapsulating messenger RNA (mRNA) for in vitro transfection, the formulation C12–200 40%/Chol. 43.5%/DOPE 15% demonstrated higher transfection efficacy towards cardiac cells, compared to the other two LNP candidates [153]. Moreover, the capacity to engineer LNPs with surface modifications enhances their specificity in targeting desired tissues, thereby further mitigating the risk of systemic side effects. To accomplish this, different strategies have been developed, such as covalent surface modification using small molecule ligands, peptides, and monoclonal antibodies. For example, Ye et al. modified mRNA‐LNPs with the fusogenic coiled‐coil peptides, which can significantly enhance the mRNA transfection for different cells, such as the difficult‐to‐transfect induced pluripotent stem‐cell‐derived cardiomyocytes (iPSC‐CMs) (Figure 9) [154]. In a murine myocardial infarction model, in vivo transfection performance was demonstrated via intramyocardial injection, providing valuable insights for cardiac‐specific delivery [154].

Polymers are macromolecules consisting of multiple repeating units known as monomers, representing another class of materials utilized for gene therapy [60, 61, 62, 63, 64, 65, 66, 67, 68, 69, 70, 71, 72, 73, 74, 75, 76, 77, 78, 79, 80, 81, 82, 83, 84, 85, 86, 87, 88, 89, 90, 91, 92, 93, 94, 95, 96, 97, 98, 99, 100, 101, 102, 103, 104, 105, 106, 107, 108, 109, 110, 111, 112, 113, 114, 115, 116, 117, 118, 119, 120, 121, 122, 123, 124, 125, 126, 127, 128, 129, 130, 131, 132, 133, 134, 135, 136, 137, 138, 139, 140, 141, 142, 143, 144, 145, 146, 147, 148, 149, 150, 151, 152, 153, 154, 155, 156]. Similar to the tunable properties within LNPs formulations, polymers offer chemical versatility, allowing precise control over their structure and functionality [155, 156, 157]. This chemical tunability enables the design of polymers with tailored characteristics, such as degradation rates, charge density, and hydrophobicity, which can be optimized for specific therapeutic applications and targeted delivery in gene therapy [95, 96, 97, 98, 99, 100, 101, 102, 103, 104, 105, 106, 107, 108, 109, 110, 111, 112, 113, 114, 115, 116, 117, 118, 119, 120, 121, 122, 123, 124, 125, 126, 127, 128, 129, 130, 131, 132, 133, 134, 135, 136, 137, 138, 139, 140, 141, 142, 143, 144, 145, 146, 147, 148, 149, 150, 151, 152, 153, 154, 155, 156, 157, 158]. Polymers with cationic charges can spontaneously condense nucleic acids via electrostatic interaction, such as poly(amidoamine) (PAMAM), polyethylenimine (PEI), and poly(β‐amino ester) (PBAE) [159, 160].

**FIGURE 10:**
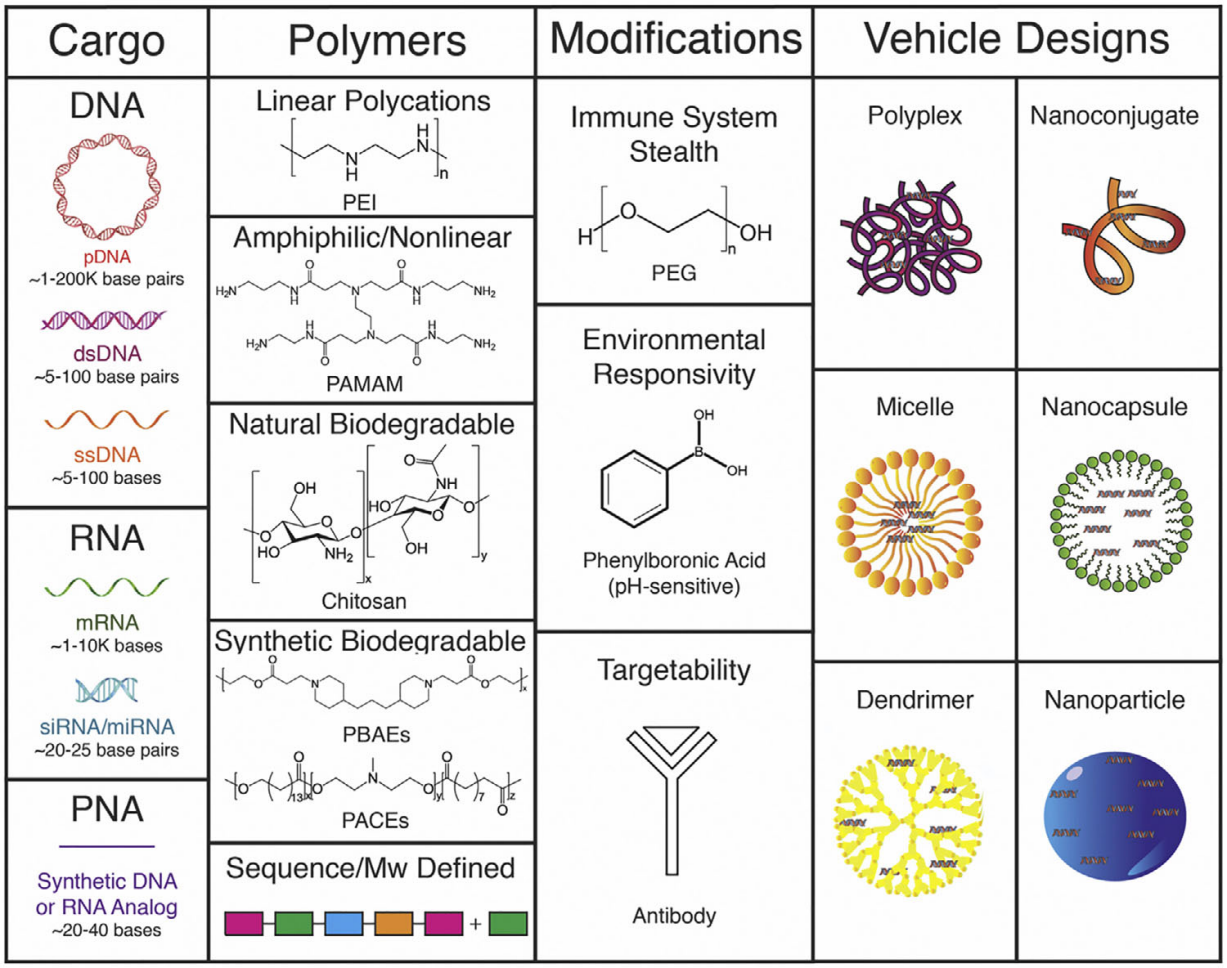
Polymeric nucleic acid delivery vehicle formulation “menu” including example choices of cargo, polymers, modifications, and end vehicle design. Vehicle designs include polyplexes‐short‐lifetime electrostatic complexes that require an excess of polymer to nucleic acids to be formed, nanoconjugates‐very short‐lifetime linear polycations with limited structure and random organization, micelles‐MW‐dependent lifetime core‐shell complex composed of dynamic amphiphilic polymers, nanocapsules—natural polymer shell complex that encapsulates cargo, dendrimer—branched polymer complex with dendritic encapsulation of nucleic acids that can have peripheral functionalized for improve delivery, and nanoparticles—solid particles with homogeneous loading of nucleic acids that require degradation to assist cargo release. Reproduced with permission [158]. Copyright 2020, Elsevier B.V.

Nevertheless, despite the general utility, emerging concerns have arisen due to the positively charged materials that can induce potential immunogenicity and cellular toxicity [161]. To address this, different strategies have been developed to minimize the cytotoxicity while maintaining high delivery efficacy [160]. A potential approach is put forth in a recent study conducted by Fang et al., wherein they suggest the substitution of the primary amino groups of PEI with neutral hydrazide groups; the resultant PEI‐based siRNA delivery nanosystem exhibited no cytotoxicity even at a high concentration of 100 µg/mL [162]. In contrast to PEI polymers, PBAE and PAMAM are other typical cationic polymer classes with improved biodegradation and reduced cytotoxicity [163]. Yet, achieving heart‐oriented delivery usually necessitates additional modifications on these polymers [42]. For instance, incorporating specific targeting ligands or using surface modifications can enhance their ability to precisely localize to cardiac tissues, which are essential to maximize the reduction of off‐target effects and improve the efficiency of gene delivery [42, 43, 44, 45, 46, 47, 48, 49, 50, 51, 52, 53, 54, 55, 56, 57, 58, 59, 60, 61, 62, 63, 64, 65, 66, 67, 68, 69, 70, 71, 72, 73, 74, 75, 76, 77, 78, 79, 80, 81, 82, 83, 84, 85, 86, 87, 88, 89, 90, 91, 92, 93, 94, 95]. As illustrated in Figure 10, within the category of polymeric delivery systems, diverse modifications and vehicle design strategies have been developed for heart‐targeted gene delivery.

Overall, lipid and polymeric nanoparticles represent the latest non‐viral platforms for gene delivery. In Table 1, we summarized the recent progress on advancing siRNA delivery by using LNPs or polymeric NPs.

**TABLE 1:** Brief summary of recent advances on LNPs and polymeric NPs‐mediated siRNA delivery in CVDs.

Vehicles	Genetic cargos	Delivery strategies	Targets	Dosing	Application	Refs.
Polymeric NPs (PLGA/DSPE‐PEG/G0‐C14)	siRNA against CaMKIIγ	i.v. injection	Macrophages	1 nmol siRNA per injection, twice a week for 4 weeks	Atherosclerosis	[78]
Lipid‐polymer NPs	siRNA against Mcp1	i.v. injection	Bone‐marrow endothelial cells	siRNA (2 mg/kg) treatment before and after surgery	Myocardial infarction	[164]
Polymer NPs (PBAEs)	siRNA against ICAM2	i.v. injection	Vascular endothelial cells	50 µg siRNA	Vascular inflammation	[165]
Biomimetic NPs (polypeptide/PC/macrophage membrane)	siRNA against Sav1	i.v. injection	Macrophages, cardiomyocytes	150 µg siRNA/kg, 10 min post‐IR injury	Myocardial IR injury	[63]
Polymer NPs (PEI‐PEG‐cRGD/PLGA NPs)	siRNA against VCAM‐1	i.v. injection	Endothelial cell	400 µg siRNA/kg, 10 min post‐IR injury	Myocardial IR injury	[166]
Polymeric NPs (modified chitosan)	siRNA against Baf60a	i.v. injection	Lesional macrophages, hepatocytes	0.2 mg/kg siRNA, twice a week for 10 weeks	Atherosclerosis	[80]
Polymeric NPs (mannose‐functionalized dendrimer)	siRNA against SR‐A	i.v. injection	Macrophages	0.1 mg/kg siRNA, weekly injection for 8 weeks	Atherosclerosis	[167]
Hybrid NPs (PEG/lipid)	siRNA against Olfr2	i.v. injection	Macrophages	1 nmol siRNA, twice per week for 4 weeks	Atherosclerosis	[168]
Zwitterionic LNPs	siRNA against PCSK9	i.v. injection	Liver	0.59 mg/kg siRNA, day 0 and day 18	Hypercholesxhbrk terolemia	[169]
LNPs	siRNA against ATP2A2	i.v. injection	Cardiomyocytes	0.4 mg/kg siRNA	apoE‐depleted animals	[170]
ECM‐LNPs	siRNA against EGFR	Intramyocardial	Leukocytes, fibroblasts	1 nmol siRNA, three injections post‐IR injury	Myocardial infarction	[171]

**Abbreviations: PLGA**, Poly(lactic‐co‐glycolic) acid; **DSPE**, 1,2‐Distearoyl‐sn‐glycero‐3‐phosphorylethanolamine; **PEG**, Polyethylene glycol; **i.v**., Intravenously injection; **PBAEs**, Poly(beta‐amino ester)s; **N/A**, Not Applicable; **PC**, poly(l‐lysine)‐cis‐aconitic acid; **IR**, Ischemic/reperfusion; **PEI**, Polyethylenimine; **cRGD**, cyclic cRGDfK peptide; **apoE**, Apolipoprotein E.

#### 4.4.2 Polysaccharide‐Based Delivery System

Polysaccharides are natural polymers defined as carbohydrate structures with repeating monosaccharides joined by glycosidic bonds [110, 111]. They represent a unique class of highly abundant biomolecules that can be obtained from natural origins, such as algae, plants, animals, and microorganisms [110, 111]. Polysaccharides are diverse in physicochemical properties depending on the chemical structures, molecular weight (MW), and chemical compositions. These identities entail them as ideal carriers for nucleic acid delivery via ionic interaction or covalent binding [172]. In this subsection, the progress on using polysaccharide‐based siRNA nanosystems is detailed below.

**FIGURE 11:**
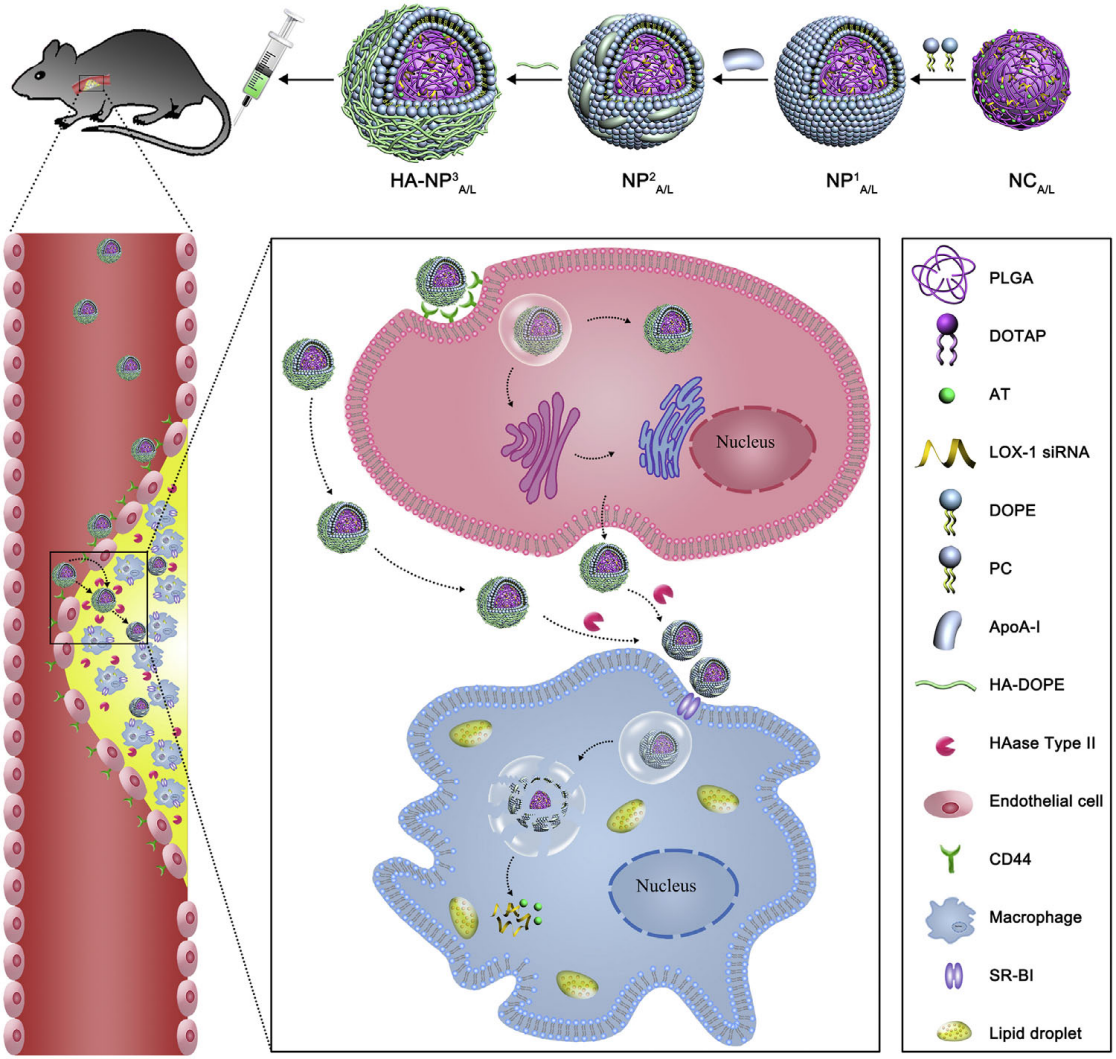
Schematic illustration of a dual‐targeting core‐shell HA‐based nanoplatform, which is designed to deliver LOX‐1 siRNA and atorvastatin (AT) to endothelial cells and macrophages in the atherosclerotic lesions selectively and sequentially. HA: hyaluronic acid. Reproduced with permission [79]. Copyright 2018, Elsevier B.V.

Precise targeting capability of siRNA nanosystems is of particular importance to gain high selectivity of RNAi machinery and avoid potential off‐target effects. Natural polysaccharides, as one of the most abundant dietary components, possess inherent properties as they can be recognized by specific lectins on certain glycans, resulting in targeted delivery without complicated modifications [111]. Among these, HA is one of the most studied carbohydrates for targeted siRNA delivery, which has shown binding affinity with the glycoprotein CD44 antigen on cell surfaces [95, 96, 97, 98, 99, 100, 101, 102, 103, 104, 105, 106, 107, 108, 109, 110, 111, 112, 113, 114, 115, 116, 117, 118, 119, 120, 121, 122, 123, 124, 125, 126, 127, 128, 129, 130, 131, 132, 133, 134, 135, 136, 137, 138, 139, 140, 141, 142, 143, 144, 145, 146, 147, 148, 149, 150, 151, 152, 153, 154, 155, 156, 157, 158, 159, 160, 161, 162, 163, 164, 165, 166, 167, 168, 169, 170, 171, 172, 173]. Due to its anionic charge, which may induce electrostatic repulsion with siRNA, most studies focused on either combinatory complexation with other polymers (e.g., poly(D,L‐lactide‐co‐glycolide) (PLGA), chitosan, and polyethylenimine (PEI)), or chemical conjugation on the backbone of HA [174, 175, 176]. For example, a core‐shell nanostructure was reported by Liu et al., where the siRNA was condensed by PLGA as the core, which was further coated with HA‐Dioleoylphosphatidylethanolamine (HA‐DOPE) as the outermost layer for dual‐targeting towards macrophage and endothelial cell (Figure 11) [79]. Biweekly in vivo administration of resultant NPs reduced plaque size by 39% and decreased lipid accumulation by 63%, as evident in a clinically relevant atherosclerotic murine model [79]. The underlying mechanisms behind plaque targeting of HA NPs were elaborated by Kim et al. In addition to the increased understanding of CD44‐binding affinity, it was shown that HA NPs can also home to stabilin‐2 (also called HARE), a receptor abundantly expressed in atherosclerotic plaques [177]. The amphiphilic HA derivatives bearing 5β‐cholanic acid were adopted to fabricate self‐assembled NPs, which were further labelled with fluorescein Sulfo‐Cyanine5.5 (Cy5.5) for in vivo visualization [177]. As a result, substantial accumulation of HA NPs was observed in plaque lesions, quantified as 1.34‐fold higher than that of control NPs [177]. Despite its inherent homing affinity towards atherosclerotic plaques, the mechanisms for heart targeting remain ambiguous. Apart from the CD44‐expressed macrophages activated in the ischemic myocardium, are there other receptors that can interact with HA NPs? For example, the Hyaluronan‐mediated motility receptor (Hmmr), a receptor for HA, was identified as a key modulator of cardiac regeneration in mammals, suggesting its potential role as a target molecule for homing nanosystems [178].

These aforementioned findings collectively indicated the feasibility of using HA‐based nanosystems for targeted siRNA delivery. Future efforts may include: (1) widening of the therapeutic window in ischemic heart diseases, such as MI, and (2) investigating the potential targets for HA binding in ischemic heart disease.

Beta‐glucans (β‐glucans) are a distinct class of polysaccharides composed of non‐starch soluble D‐glucose monomers connected by β‐glycosidic bonds [179]. They are widely distributed in yeast, fungi (including mushrooms), certain bacteria, seaweeds, and cereals such as oats and barley [179]. Owing to their intrinsic interaction with bioactive molecules, β‐glucan‐based nanocarriers have received considerable attention in recent years [180]. The binding affinity of β‐glucan recognition likely depends on the cell types involved and the receptors engaged [181]. For example, complement receptor 3 (CR3) is an integrin dimer highly expressed in myeloid cells, which can interact with β‐glucan via “lectin domain” and contribute to coordinate clustering of other low‐affinity receptors [181]. Moreover, the “lectin domain” may facilitate interactions with other membrane components, such as lactosylceramide, which is likely an essential partners in recognizing β‐glucan [181].

**FIGURE 12:**
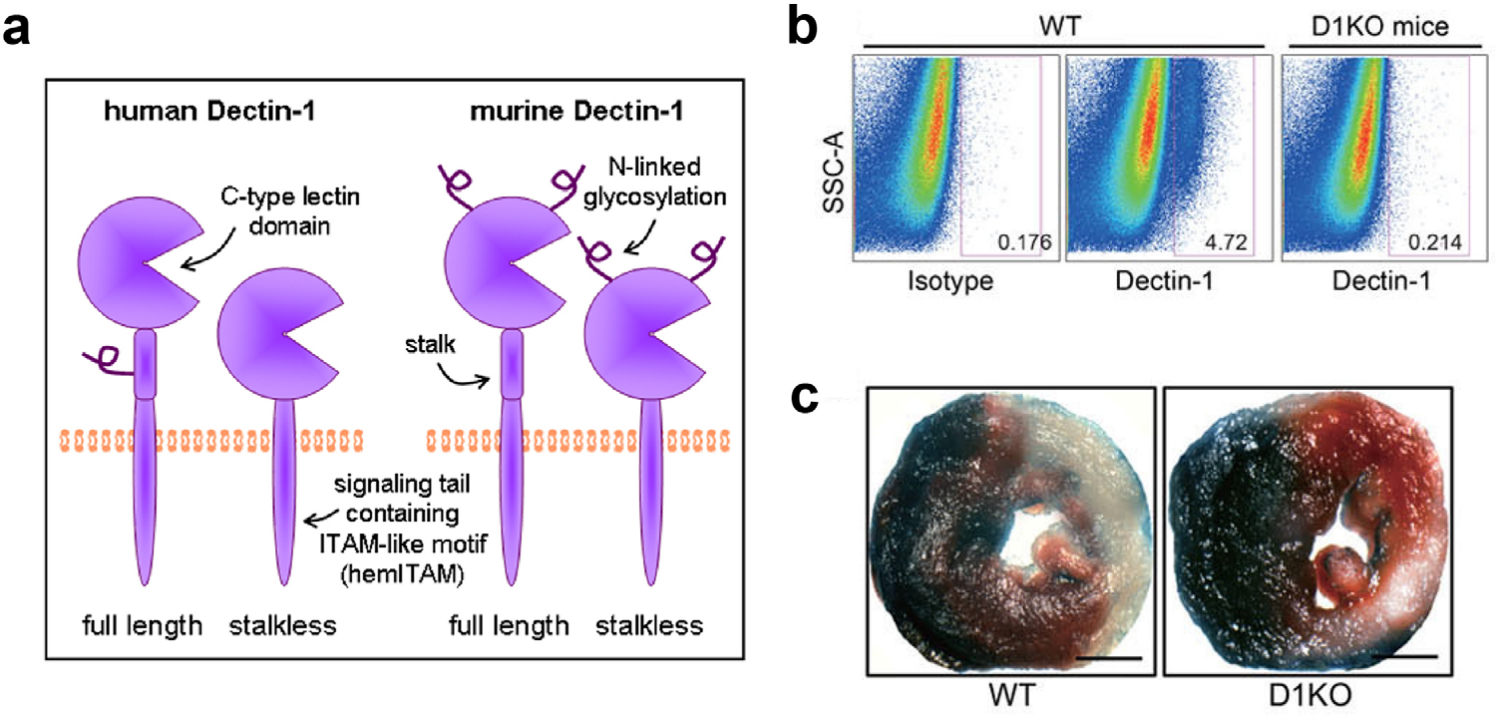
(a) Dectin‐1 consists of an extracellular C‐type lectin domain for β‐(1,3)‐glucan detection, linked via a transmembrane domain to an intracellular signaling tail. Adapted and reprinted with permission from ref [181]. Copyright 2009, John Wiley & Sons. (b) Flow cytometric analyses of Dectin‐1 expression on cardiac immune cells isolated from both wild‐type (WT) and Dectin‐1 knockout (D1KO) mice at day 1 after IR. (c) Left ventricular (LV) tissue sections of both WT and D1KO mice stained with Evans blue and 2,3,5‐triphenyltetrazolium chloride at day 2 after IR to delineate the area at risk (AAR) and the infarcted region (scale bar, 1 mm). Reproduced with permission [188]. Copyright 2018, Wolters Kluwer Health.

Among various phagocytic receptors, Dectin‐1 is the most extensively studied for recognizing β‐glucan via the canonical phagocytosis process [182]. Dectin‐1 is a natural killer (NK)‐cell‐receptor‐like C‐type lectin that is primarily expressed on cells of myeloid origin, including macrophages, monocytes, neutrophils, and a subset of B and T lymphocytes [181, 182, 183]. It has been reported that Dectin‐1 has a carbohydrate recognition domain (CRD) consisting of six cysteine residues that are highly conserved in C‐type lectins, in which Trp [184] and His [185] are critical residues for the specific binding of β‐glucan (Figure 12a) [181, 182, 183, 186]. Moreover, in the seminal work by Gordon et al., Dectin‐1 was identified as the predominant receptor for the nonopsonic recognition of β‐glucan‐based particles [187]. These findings illustrated the role of Dectin‐1 receptor involved in the innate immune responses and provided insights into the rational design of β‐glucan‐based drug delivery systems. More recently, Dectin‐1‐expressing macrophage subsets have been identified as pathological mediators of myocardial IR injury, underscoring their potential as significant therapeutic targets (Figure 12b,c) [188]. From a pharmaceutical standpoint, development of β‐glucan‐based nanosystems tailored to target Dectin‐1 represents a promising avenue to advance RNAi therapeutics in the realm of cardiac therapy.

In our group, different β‐glucan‐based nanoformulations have been fabricated for drug delivery applications. One study conducted by Liu et al. demonstrated that co‐assembly of chitosan with β‐glucan resulted in the formation of biocompatible NPs, which were capable of inhibiting NLRP3 inflammasome when combined with small molecule CY‐09 in a Dectin‐1‐dependent manner [189]. Targeted delivery of siRNAs towards specific organs is of particular importance to maximize gene silencing efficacy and avoid premature off‐target effects; yet, current strategies relied on external components’ modifications may require multiple fabrication steps and thereby weaken the stability of RNA [95]. To address this, one recent work from our group proposed a “targeting‐ligand‐free” strategy for tailored siRNA delivery [138]. To optimize the intracellular release of siRNA, β‐glucan‐based polysaccharide was further conjugated with a virus‐mimicking endosomolytic poly(alkyl methacrylate‐co‐methacrylic acid) fragments through a reversible addition‐fragmentation chain transfer (RAFT) polymerization method [138]. The resultant CEEPG NPs showed pH‐dependent hemolytic activities with satisfactory endosomal escape capabilities, leading to efficient gene silencing efficacy in distinct inflammatory scenarios, including MI, lung fibrosis, and liver inflammation [138].

Overall, the above‐mentioned findings suggested the feasibility of developing β‐glucan‐based nanoformulations for drug delivery applications, holding great potential for resolving advanced inflammation progression in multiple diseases.

#### 4.4.3 Exosome‐Based Therapy

Small extracellular vesicles (Exosomes, EVs) are nanosized vesicles that are naturally secreted by biological systems. Instead of using cells for restoring the dysfunction of the heart after ischemia, cell‐free‐based delivery has drawn great interest in the field of cardioprotection [190, 191]. Exosomes are present in all body fluids and have been involved in nearly all biological and pathogenic processes [192]. In particular, these nanoscale extracellular vesicles can manipulate intercellular interactions by transferring biological molecules and targeting acceptor cells [193, 194]. These properties enable them to play important roles in the maintenance of tissue homeostasis. In this subsection, we go through the exosome‐based therapeutic formulations for heart regeneration.

The use of native EVs as therapeutic interventions for cardiovascular diseases has been exploited over the past decades [195, 196]. Given that EVs from different cellular resources may differ regarding the regulating mechanisms for cardiac regeneration [197], we focus on discussing the cardioprotective effects of EVs upon their cellular origins, specifically within the pathological contexts of MI, atherosclerosis, myocardial fibrosis, and heart failure. To date, the mesenchymal stem cells (MSCs) derived exosomes are the most widely investigated therapeutics for cardiac remodeling [198, 199]. As exosomes can carry the cellular components from their parent cells, among which the miRNAs are mostly studied in cardiovascular repair [200]. Depending on the miRNA contents of EVs, they can trigger different cardioprotective effects, including anti‐inflammatory [201, 202], activation of T cell‐based immune regulation [203], stimulation of angiogenesis [202, 203, 204], attenuation of cardiac fibrosis [205, 206, 207], and regulation of cell death programs [208]. Most recently, researchers revealed the pathological role of miR‐200b‐3p carried in MSC‐derived EVs, which can decrease apoptosis and inflammatory responses by deactivating the NLR family pyrin domain containing 1 (NLRP1) inflammasome upon targeting BCL2L11 (Bcl‐2‐like protein 11) [209].

In addition to MSCs, exosomes generated/derived from induced pluripotent stem cells (iPSCs) have been shown to have potential clinical translation properties in human heart diseases [210]. In particular, as a cell‐free therapy, iPSCs‐EVs may replicate bioactive functions of iPSCs with less immunogenicity and tumorigenicity [210, 211]. A comprehensive study conducted by Bobis‐Wozowicz et al. demonstrated that iPSC‐EVs carry numerous functional molecules, including proteins, miRNAs, and mRNAs [212]. By comparing with their cellular counterparts, the iPSCs‐EVs were shown to contain more proteins that are involved in receptor binding, signal transduction, and regulation of biological processes [212]. Of note, pluripotency‐associated factors were shown to be particularly enriched in iPSCs‐EVs, which is essential for the maintenance of pluripotency [212]. Subsequently, the exosomal miRNA compositions from iPSCs and 3 major iPSC‐derived cardiac cell types were analyzed, in which the miRNAs were shown to have distinct expression profiles based on the original cell type [213]. For example, miR‐1 was only secreted by iPSC‐derived cardiomyocytes, whereas miR‐155 was primarily secreted in iPSC‐derived cardiac fibroblasts [213]. These studies provided useful information towards defining the expression pool of biological molecules from iPSCs‐EVs, which encourages further investigation into exosomes that are generated from specific cell types.

**FIGURE 13:**
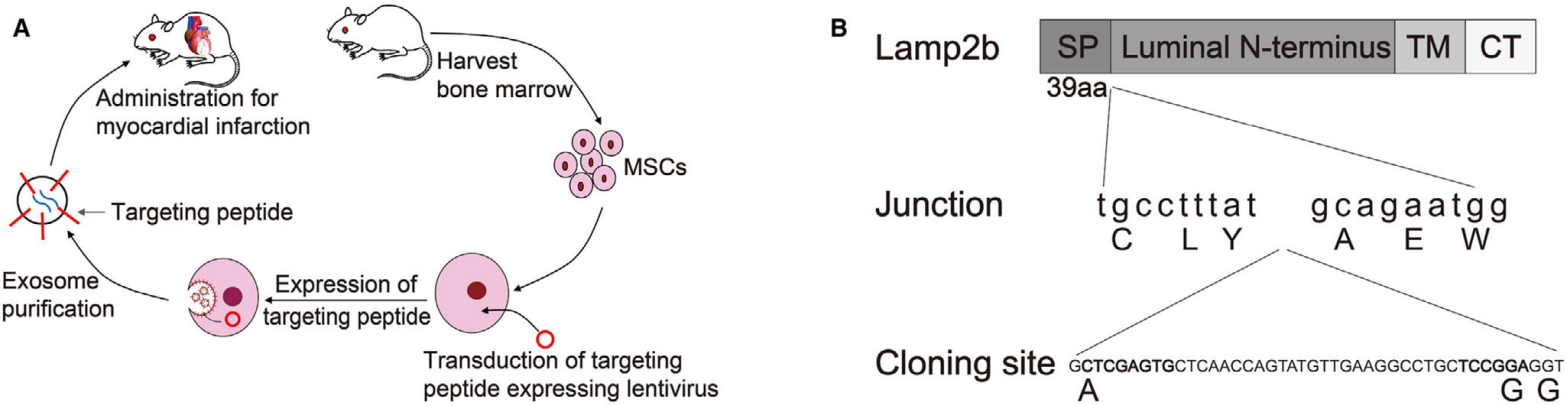
Reconstruction of exosomes exhibited with ischemic myocardium‐targeting peptide CSTSMLKAC fused with Lamp2b. (a) Schematic diagram of production, purification, and systematic injection of targeted exosomes for myocardial infarction therapy. (b) Schematic diagram of the re‐administration of Lamp2b protein. CT indicates C terminus; MSC, mesenchymal stem cell; SP, signal peptide; TM, transmembrane domain. Reproduced with permission [215]. Copyright 2018, John Wiley and Sons.

However, the clinical potential of native EVs for cardiovascular therapy is limited due to their short half‐life, off‐target cellular uptake, and high clearance [214]. Therefore, additional investigations were performed to augment the therapeutic efficacy of exosomes in the treatment of cardiovascular diseases. In this case, different bioengineering technologies were explored to increase the bioactivity and targeting capability of exosomes. One common strategy to enhance internalization is to fuse exosomal membrane proteins with ligands or homing peptides. To achieve this, Xu Wang et al. reprogrammed MSCs‐EVs by fusing with ischemic myocardium‐targeting peptide IMTP (CSTSMLKAC), resulting in a 16.1% decrease in cardiac fibrosis length compared with the blank exosomes (Figure 13) [215]. Improved left ventricular ejection fraction was also found in the IMTP‐modified EVs group [215]. In another approach, the EVs‐secreting cells can be modified by transfecting with exogenous molecules, such as plasmid DNA or miRNAs [216]. For example, cardiosphere‐derived cells (CDCs) were genetically transfected with cardiomyocyte‐specific binding peptide (CMP), which can further secrete exosomes with the peptide of interest on their surface [216]. As a result, the CMP‐targeted exosomes showed significantly enhanced cardiac tropism, with a fold change of 4.3 ± 1.0 vs. control CDC‐EVs (1.9 ± 0.5) [216]. Similar strategies were also reported elsewhere [217]. Nevertheless, further investigations should focus on the potential therapeutic efficacy, scale‐up feasibility, and in vivo safety of these bioengineered exosomes.

#### 4.4.4 From Bench to Bedside: Translational Status, Challenges, and Future Perspectives

Despite progress in nanoparticle design for targeted cardiovascular therapies, several limitations persist that impact their clinical translation. One major challenge is achieving effective targeting specifically to cardiac tissues. Developing and optimizing ligands that can selectively bind to cardiac cells, avoiding interactions with the protein corona, and determining the optimal ligand valency remain critical obstacles [218, 219, 220]. These issues can lead to reduced targeting efficacy and increased off‐target effects, thereby hindering the clinical translation of nanoparticles designed for cardiac applications.

The role of the protein corona, proteins that adhere to nanoparticles in biological fluids, is especially pertinent in cardiac targeting [184]. The composition of the protein corona can influence the biodistribution and cellular uptake of nanoparticles, affecting their ability to accumulate in heart tissues and exert therapeutic effects [221]. Recent studies have underscored the need for detailed mechanistic research to better understand how specific corona proteins interact with nanoparticles and influence their cardiac delivery [185, 222].

These challenges also present significant opportunities for improvement. For instance, active targeting could be achieved by leveraging the pathological features of the disease microenvironment, such as the decoration with cardiac homing peptides and conjugation with antibodies [223, 224, 225]. Under ischemic conditions, tight junctions in cardiac capillary endothelium are disrupted, which may allow for passive transportation of nanoparticles [226]. By further investigating the mechanistic factors influencing cardiac nanoparticle delivery and refining strategies for both passive and active targeting, researchers can enhance the design and effectiveness of nanoparticles for heart‐tropic treatments. Advances in this area could lead to more precise and effective therapies for cardiovascular diseases, improving patient outcomes and paving the way for successful clinical applications.

Over the past decade, promising findings from numerous publications have led to a number of ongoing clinical investigations. Table 2 summarizes the latest progress on clinical trials using genetic therapeutics for the treatment of CVDs.

**TABLE 2:** Summary of clinical trials investigating genetic therapeutics for the treatment of CVDs.

Genetic therapeutics	Delivery strategies	Condition or disease	Trial name (number)	Drug target	Trial design	Primary endpoint	Outcome	Status
ASOs	Ligand‐conjugated^a^ antisense (LICA)	CVDs Elevated Lp [a]	NCT04023552	lipoprotein(a) [Lp(a)]	Phase III	Time to first MACE	Results not yet reported	Estimated completion (02/2026)
ASOs	Synthetic ASOs with a fully phosphorylated backbone	Myocardial infarction	NCT05350969	miR‐132	Phase II	Left ventricular end‐systolic volume (LVESVI) measured by ECHO	Results not yet reported	Completed (03/2025)
siRNA	N‐acetyl galactosamine–conjugated siRNA	Atherosclerosis Elevated Lp [a]	NCT04270760	lipoprotein(a) (Lp[a])	Phase II	Changes in the level of Lp[a]	Achieved >95% Lp(a) reduction; supporting transition to Phase 3	Completed (11/2022)
siRNA	GalNAc‐conjugated siRNA	Atherosclerosis Elevated LDL‐Cholesterol	NCT04929249	PCSK9	Phase III	Changes in the level of LDL‐C	60% LDL‐C reduction; supporting an “Inclisiran‐first” strategy for frontline clinical translation	Completed (09/2023)
siRNA	GalNAc‐conjugated siRNA	Atherosclerosis Elevated Lp [a]	NCT04606602	lipoprotein(a) (Lp[a])	Phase I	Incidence of treatment‐emergent adverse events	Results not yet reported	Completed (08/2023)
siRNA	Lipid nanoparticles	Elevated LDL‐Cholesterol	NCT01437059	PCSK9	Phase I	Safety, tolerability and Changes in the level of LDL‐C	Reduced LDL cholesterol concentration	Completed (09/2012)
siRNA	Lipid nanoparticles	Transthyretin Amyloidosis with Cardiomyopathy	NCT03997383	Hepatic transthyretin (TTR)	Phase III	Safety and efficacy	Completed 12‐month efficacy phase with preserved functional capacity; OLE ongoing	Estimated completion (03/2027)
siRNA	GalNAc‐conjugated siRNA	Transthyretin Amyloidosis with Cardiomyopathy	NCT04153149	Transthyretin (TTR)	Phase III	Safety and efficacy	Met primary/secondary endpoints, 28% reduction in MACE; OLE ongoing	Estimated completion (12/2026)
DNA plasmid	DNA plasmid	Acute Myocardial Infarction	NCT03404024	Hepatocyte growth factor (HGF)	Phase II	Safety, tolerability and LVEF measured by cardiac MRI	Results not yet reported	Completed (08/2019)
mRNA	Citrate‐buffered saline	Heart Failure	NCT03370887	Vascular Endothelial Growth Factor (VEGF‐a)	Phase II	Safety and tolerability	Met primary endpoint, limited sample size for clinical translation	Completed (06/2021)
mRNA	Lipid nanoparticles	Heart Failure	NCT05659264	Relaxin	Phase I	Safety and tolerability	Results not yet reported	Completed (12/2024)

^a^ **Abbreviations: ASOs**, Antisense oligonucleotides; **CVDs**, Cardiovascular diseases; **LDL**, Low density lipoprotein; **MACE**, Major adverse cardiovascular events; **PCSK**9, Proprotein convertase subtilisin kexin 9; **siRNA**, Small interfering RNA; **mRNA**, Messenger RNA; **GalNAc**, Triantennary N‐acetylgalactosamine carbohydrates; **OLE**, Open label extension.

## 5 Conclusions and Future Perspectives

Nanomedicine holds immense promise as a paradigm‐shifting approach in reshaping the landscape of cardiovascular therapy. Despite substantial progress in improving tissue targeting and payload stability, the path toward clinical reality remains fraught with challenges. Bridging the gap necessitates surmounting existing challenges and exploring emerging frontiers. This section outlines the current limitations in cardiac nanotherapeutics, highlights potential opportunities, and explores how computational tools will orchestrate the development of next‐generation platforms for precision cardiology.

### 5.1 Limitations and Opportunities

Despite the remarkable potential of RNAi nanotherapeutics across the CVD spectrum, the transition from controlled laboratory environment to the clinic remains a formidable challenge. Current cardiac nanomedicine may show efficacy in rodent models that suffer myocardial ischemia or atherosclerosis, whereas these overlook the inter‐patient heterogeneity observed in clinical settings, such as the variations in age and gender‐dependent dynamics of the immune response. The “biological identity” of the nanomaterials, governed by the formation of protein corona, remains inadequately understood under the high‐shear hemodynamic conditions of the heart. Surface modifications of delivery systems may optimize the tissue targeting and immune reactivity, whereas such strategies also raise translational concerns, including batch‐to‐batch reproducibility, scale‐up potentialities, and safety issues. From the perspective of RNAi nanotherapeutics, striking a balance between nanosystems’ efficacy with relatively low‐dose, efficient cytosolic release of RNA payloads, and long‐term stability is imperative for personalized cardiac therapy. Furthermore, a notable gap in current literature is the lack of standardized quantification for cardiac homing efficiency; some studies rely on qualitative imaging rather than quantitative metrics to define heart tropism, which may pose challenges for interpretability and cross‐platform comparisons.

These limitations also delineate potential opportunities for innovation. A deeper mechanistic understanding of nano‐bio interactions in pathological cardiac microenvironments may enable more rational design of delivery systems with improved specificity and intracellular trafficking. Harnessing the power of the immune system may allow for smart nanosystem development that leverages and manipulates the pathogenic process. More importantly, researchers are encouraged to utilize transformative tools to accelerate the rational design frameworks. For instance, the emergence of artificial intelligence (AI) approaches offers promising avenues to refine nanoparticles' performance, allowing for efficient formulation prediction and screening that was not possible prior to conventional “trial‐and‐error” approaches. In addition, integrating multi‐omics data from cardiac patients with in silico analysis may boost the era of AI‐driven personalized cardiac nanomedicine.

### 5.2 Computational‐Aided Rational Design

As illustrated above, AI is transforming the landscape of nanomedicine toward smart, precision, and accurate therapy. In this section, we showcase the potentialities of combining computational tools for assisting nanoparticle design, with an emphasis on the application of machine learning and in silico simulations. While specific applications in cardiovascular therapy are currently emerging, pioneering studies across the nanomedicine landscape provide proof‐of‐concept insights that are detailed below.

#### 5.2.1 Machine Learning

Tuning the physicochemical parameters of delivery vehicles, such as shape, composition, and stiffness, can significantly impact their efficacy in transporting therapeutic agents, emphasizing a rational strategy for NPs design and strategy. Machine learning (ML), a subset of artificial intelligence, presents new opportunities to integrate nanotechnology with computer science, facilitating the development of next‐generation nanotherapeutics [227]. Via integrating certain substantial datasets, ML can be utilized for performing different tasks, for example, prediction and clustering [227]. In a recent study, to expedite ionizable cationic lipid discovery for mRNA delivery, Li et al. reported a screening strategy that combines machine learning and high‐throughput synthesis [228]. Using two combinatorial lipid libraries, a total of 584 LNPs were synthesized with structures identified, including in vitro and in vivo transfection efficacy, providing a foundational dataset for subsequent ML [228]. Starting from lipid chemical structures, encoded with the PaDEL‐Descriptor, the authors assessed three nonlinear ML algorithms (random forest, logistic regression, and gradient boosting), of which the XGBoost model outperformed the others and was selected as the final predictive model [228]. Following the ML‐based predictions, a new library of 40 000 untested lipids was generated, allowing for deeper evaluations of the top candidates [228]. In line with this, the out‐of‐batch lipids exhibited better performance compared to commercial benchmarks (Dlin‐MC3‐DMA and SM102), underscoring the feasibility and efficiency of the developed streamlined approach for nanoformulations’ screening [228]. While ML approaches typically require large datasets for learning to be effective, recent advancements enable researchers to leverage the limited size of a dataset for ML tasks, such as the Bayesian [227, 228, 229]. One study led by WAHL et al., via leveraging Bayesian optimization (BO), proposed an ML‐driven, closed‐loop integration process for unveiling the relationship between physical compositions and targeting property of the NPs [230]. With this platform, they can synthesize new and more complex biphasic NPs with success rates far greater than random selection (18 of 19), indicative of the great promise of ML‐accelerated design of multimetallic nanomaterials [230].

Beyond guiding the design and synthesis of nanoparticles, the distinctive advantages of machine learning enable the identification of key variables that govern nano‐bio interactions [231]. For example, to probe the structure‐function relationship of NPs towards cellular compartments, a pooled screening combined multiomic annotation and ML was developed [232]. Coupling *k*‐means clustering with biomarker findings, the constructed genomic interaction networks identified the gene SLC46A3 negatively regulated liposome NP uptake, underscoring the potential of ML‐driven screening and interpretation of NPs engagement associated with cellular patterns [232]. Moreover, understanding the interactions between NPs and tissues at the desired site is critical for the carrier's design and improving the delivery efficacy. With the aid of image‐segmentation‐based ML technology (nano‐ISML), the heterogeneous nature of vascular permeability in different tumors was characterized, leading to the tailored design of nanoparticles with improved transendothelial transport [233]. For a comprehensive and in‐depth discussion on the machine learning‐assisted interpretation of nano‐bio interactions, the reader is encouraged to consult previous reviews dedicated to this subject [231, 232, 233, 234]. However, compared with cancer nanomedicine, few studies have incorporated the desired delivery profile into a computer algorithm in the paradigm of CVDs, highlighting an immense design space for advancing targeted cardiac delivery strategies.

#### 5.2.2 Computational Simulations

Beyond taking advantage of machine learning to consolidate data into a valuable dataset and aid in the interpretation and understanding of the nanoparticles' behavior, combining computational simulation methods holds potential opportunities to accelerate and improve the nanomedicine development processes. One of the most commonly used strategies is molecular dynamics (MD) simulations, which can predict key properties of nanomaterials at the atomic level, such as surface energy, stability, and interaction with biological molecules [235, 236]. In a study by Fairen‐Jimenez et al., MD simulations were applied to identify the optimal porous metal‐organic framework (MOF) structure that was compatible with the siRNA molecule, simultaneously uncovering the energetic basis of siRNA binding within the confined pore channels [237]. Following the initial screening, a Zr_6_‐based MOF (termed as nNU‐1000) was selected, with thermodynamic analyses confirming the feasibility of siRNA encapsulation within its 3 nm pores [237]. Furthermore, by leveraging MD simulation, the internal binding energy was revealed, indicating a favorable packing process (−878 kJ/mol) without inducing conformational strain, allowing for the structural integrity as a gene delivery system [237]. By providing a molecular‐level map of MOF‐siRNA interactions, this computationally‐driven approach offers a predictive framework that facilitates the precise engineering of advanced nanocarriers for gene therapy.

The effect of protein corona on nanomaterials exposes with endogenous biological environments is of paramount importance, which can dictate their safety and biodistribution [185, 222, 223, 224, 225, 226, 227, 228, 229, 230, 231, 232, 233, 234, 235, 236, 237, 238]. To deconvolute the complexities of corona buildup and complement the limitations of traditional experiments, one suitable strategy is the MD‐based simulations at both atomistic and coarse‐grained levels [239, 240]. Recent work by Wei et al. harnessed multiscale simulations to investigate the protein corona formed on the surface of silver nanoparticles (AgNPs) [240]. Major factors, including surface hydrophilicity, size, and curvature, were comprehensively analyzed via the combination of coarse‐grained molecular dynamics (CGMD) and atomistic simulations [240]. Mesoscopic CGMD simulations revealed different microscopic kinetics of peptide adsorption on AgNPs, progressing through three stages: initial landing on hydrophilic surfaces, increased surface contact driven by silver‐water interactions, and structural denaturation altering surface residue distribution [240]. As NP size decreases, peptides exhibit more adsorption‐desorption dynamics, with higher packing density and greater orientational distribution on smaller NPs [240]. This analytical strategy identifies unique fingerprints on protein corona formation dynamics and peptides’ structural changes, thereby guiding hydrophilic NPs’ design towards complex biological environments.

Surface modification is a critical component in tailoring nanoparticles for targeted cell/tissue delivery, while distinct decorating strategies may elicit varying influences on intracellular interactions [241, 242]. To gain deeper insights into the tuning conjugation strategies towards uptake dynamics, Ma et al. investigated the underlying mechanisms of NPs’ cellular uptake process under different physiological conditions, with the aid of dissipative particle dynamics (DPD) simulation [243]. The binding strengths of varied ligands on gold nanoparticles were quantified using semiempirical quantum mechanics (SQM) and density functional theory (DFT), demonstrating distinct binding energies as a result of anchoring moieties [243]. With DPD simulation, the authors further explored the relationship between binding strengths and cellular uptake. They observed that during cellular uptake, ligand‐coated nanoparticles with N_3_‐terminated conjugation (28.0 *k_B_T*) exhibit uneven ligand distribution (λ ≈ 0.60) as receptors diffuse to facilitate membrane wrapping, leading to partial engulfment [243]. In contrast, nanoparticles with a pattern distribution of ligands showed enhanced cellular uptake compared to uniform ligand distribution, indicating the importance of receptor‐ligand (R‐L) specific interactions [243]. By integrating experimental work and computational simulations, this study offers specific guidance on tailored nanoparticle design and optimization.

### 5.3 Conclusions

In summary, this review provides a state‐of‐the‐art overview of RNAi nanotherapeutics in the treatment of CVD, centering on the applications in myocardial infarction and atherosclerosis. By critically assessing the transition from experimental platforms to clinical‐ready solutions, we underscore that the transformative frontier in cardiovascular nanomedicine lies in the synergy between innovative delivery materials and data‐driven smart design. Looking forward, we envision that the convergence of multidisciplinary expertise—bridging material science, cardiology, and computational intelligence—will facilitate personalized strategies for advancing next‐generation RNAi cardiac nanomedicine.

## Conflicts of Interest

The authors declare no conflicts of interest.

## Data Availability

The authors have nothing to report.

## References

[1] A. Doku , L. S. Tuglo , V. Boima , et al., “Prevalence of Cardiovascular Disease and Risk Factors in Ghana: a Systematic Review and Meta‐analysis,” Global Heart 19 (2024): 21, 10.5334/gh.1307. 38404614 PMC10885824

[2] S. S. Martin , A. W. Aday , Z. I. Almarzooq , et al., “2024 Heart Disease and Stroke Statistics: A Report of US and Global Data From the American Heart Association,” Circulation 149 (2024): 347, 10.1161/CIR.0000000000001209. PMC12146881 38264914

[3] A. R. Folsom , H. Yatsuya , J. A. Nettleton , P. L. Lutsey , M. Cushman , and W. D. Rosamond , “Community Prevalence of Ideal Cardiovascular Health, by the American Heart Association Definition, and Relationship with Cardiovascular Disease Incidence,” Journal of the American College of Cardiology 57 (2011): 1690–1696, 10.1016/j.jacc.2010.11.041. 21492767 PMC3093047

[4] R. A. Byrne , X. Rossello , J. J. Coughlan , et al., “2023 ESC Guidelines for the Management of Acute Coronary Syndromes,” European Heart Journal 44 (2023): 3720–3826, 10.1093/eurheartj/ehad191. 37622654

[5] M. Litviňuková , C. Talavera‐López , H. Maatz , et al., “Cells of the Adult human Heart,” Nature 588 (2020): 466–472, 10.1038/s41586-020-2797-4. 32971526 PMC7681775

[6] A. V. Kristen , S. Ajroud‐Driss , I. Conceição , P. Gorevic , T. Kyriakides , and L. Obici , “Patisiran, an RNAi Therapeutic for the Treatment of Hereditary Transthyretin‐Mediated Amyloidosis,” Neurodegenerative Disease Management 9 (2019): 5–23, 10.2217/nmt-2018-0033. 30480471

[7] A. Akinc , M. A. Maier , M. Manoharan , et al., “The Onpattro Story and the Clinical Translation of Nanomedicines Containing Nucleic Acid‐based Drugs,” Nature Nanotechnology 14 (2019): 1084–1087, 10.1038/s41565-019-0591-y. 31802031

[8] E. J. Anderson , N. G. Rouphael , A. T. Widge , et al., “Safety and Immunogenicity of SARS‐CoV‐2 mRNA‐1273 Vaccine in Older Adults,” New England Journal of Medicine 383 (2020): 2427, 10.1056/NEJMoa2028436. 32991794 PMC7556339

[9] B. R. Smith and E. R. Edelman , “Nanomedicines for Cardiovascular Disease,” Nature Cardiovascular Research 2 (2023): 351–367, 10.1038/s44161-023-00232-y. PMC13297491 39195953

[10] A. E. Eldeeb , S. Salah , and N. A. Elkasabgy , “Biomaterials for Tissue Engineering Applications and Current Updates in the Field: a Comprehensive Review,” Aaps Pharmscitech [Electronic Resource] 23 (2022): 267, 10.1208/s12249-022-02419-1. 36163568 PMC9512992

[11] V. J. Dzau and C. P. Hodgkinson , “RNA Therapeutics for the Cardiovascular System,” Circulation 149 (2024): 707–716, 10.1161/CIRCULATIONAHA.123.067373. 38408142

[12] D. Bumcrot , M. Manoharan , V. Koteliansky , and D. W. Y. Sah , “RNAi Therapeutics: a Potential New Class of Pharmaceutical Drugs,” Nature Chemical Biology 2 (2006): 711–719, 10.1038/nchembio839. 17108989 PMC7097247

[13] M. Moazzam , M. Zhang , A. Hussain , X. Yu , J. Huang , and Y. Huang , “The Landscape of Nanoparticle‐based siRNA Delivery and Therapeutic Development,” Molecular Therapy 32 (2024): 284–312, 10.1016/j.ymthe.2024.01.005. 38204162 PMC10861989

[14] H. Gao , R. Cheng , and H. A. Santos , β‐glucan‐based nanosystems for targeted delivery of RNAi therapeutics for cardiac therapy (University of Groningen, 2025), 20200111, 10.33612/diss.1166453018.

[15] K. K. Ray , R. S. Wright , D. Kallend , et al., “Two Phase 3 Trials of Inclisiran in Patients with Elevated LDL Cholesterol,” New England Journal of Medicine 382 (2020): 1507, 10.1056/NEJMoa1912387. 32187462

[16] A. Fire , D. Albertson , S. W. Harrison , and D. G. Moerman , “Production of Antisense RNA Leads to Effective and Specific Inhibition of Gene Expression in C. elegans Muscle,” Development (Cambridge, England) 113 (1991): 503–514, 10.1242/dev.113.2.503. 1782862

[17] A. Fire , S. Xu , M. K. Montgomery , S. A. Kostas , S. E. Driver , and C. C. Mello , “Potent and Specific Genetic Interference by Double‐stranded RNA in Caenorhabditis elegans,” Nature 391 (1998): 806–811, 10.1038/35888. 9486653

[18] R. W. Carthew and E. J. Sontheimer , “Origins and Mechanisms of miRNAs and siRNAs,” Cell 136 (2009): 642–655, 10.1016/j.cell.2009.01.035. 19239886 PMC2675692

[19] R. L. Setten , J. J. Rossi , and S.‐P. Han , “The Current state and Future Directions of RNAi‐based Therapeutics,” Nature Reviews Drug Discovery 18 (2019): 421–446, 10.1038/s41573-019-0017-4. 30846871

[20] G. Tang , “siRNA and miRNA: an Insight into RISCs,” Trends in Biochemical Sciences 30 (2005): 106–114, 10.1016/j.tibs.2004.12.007. 15691656

[21] J. K. W. Lam , M. Y. T. Chow , Y. Zhang , and S. W. S. Leung , “siRNA versus miRNA as Therapeutics for Gene Silencing,” Molecular Therapy—Nucleic Acids 4 (2015): 252, 10.1056/NEJMoa191238710.1038/mtna.2015.23. PMC4877448 26372022

[22] G. Meister and T. Tuschl , “Mechanisms of Gene Silencing by Double‐stranded RNA,” Nature 431 (2004): 343–349, 10.1038/nature02873. 15372041

[23] S. P. R. Romaine , M. Tomaszewski , G. Condorelli , and N. J. Samani , “MicroRNAs in Cardiovascular Disease: an Introduction for Clinicians,” Heart 101 (2015): 921–928, 10.1136/heartjnl-2013-305402. 25814653 PMC4484262

[24] P. Landgraf , M. Rusu , R. Sheridan , et al., “A Mammalian microRNA Expression Atlas Based on Small RNA Library Sequencing,” Cell 129 (2007): 1401–1414, 10.1016/j.cell.2007.04.040. 17604727 PMC2681231

[25] S.‐S. Zhou , J.‐P. Jin , J.‐Q. Wang , et al., “miRNAS in Cardiovascular Diseases: Potential Biomarkers, Therapeutic Targets and Challenges,” Acta Pharmacologica Sinica 39 (2018): 1073–1084, 10.1038/aps.2018.30. 29877320 PMC6289363

[26] R. A. Boon and S. Dimmeler , “MicroRNAs in Myocardial Infarction,” Nature Reviews Cardiology 12 (2015): 135–142, 10.1038/nrcardio.2014.207. 25511085

[27] S. K. Gupta , A. Foinquinos , S. Thum , et al., “Preclinical Development of a MicroRNA‐Based Therapy for Elderly Patients with Myocardial Infarction,” Journal of the American College of Cardiology 68 (2016): 1557–1571, 10.1016/j.jacc.2016.07.739. 27687198

[28] O. Gidlöf , P. Andersson , J. van der Pals , M. Götberg , and D. Erlinge , “Cardiospecific microRNA Plasma Levels Correlate with Troponin and Cardiac Function in Patients with ST Elevation Myocardial Infarction, Are Selectively Dependent on Renal Elimination, and Can Be Detected in Urine Samples,” Cardiology 118 (2011): 217–226, 10.1159/000328869. 21701171

[29] M. Marketou , J. Kontaraki , E. Zacharis , et al., “Peripheral Blood MicroRNA‐21 as a Predictive Biomarker for Heart Failure with Preserved Ejection Fraction in Old Hypertensives,” American Journal of Hypertension 37 (2023): 298–305, 10.1093/ajh/hpad109. 37976292

[30] A. Gallo , V. Agnese , S. Sciacca , et al., “MicroRNA‐30d and ‐483‐3p for Bi‐Ventricular Remodelling and miR‐126‐3p for Pulmonary Hypertension in Advanced Heart Failure,” ESC Heart Failure 11 (2024): 155–166, 10.1002/ehf2.14546. 37864482 PMC10804158

[31] M. Węgiel , M. Surmiak , K. P. Malinowski , et al., “In‐Hospital Levels of Circulating MicroRNAs as Potential Predictors of Left Ventricular Remodeling Post‐Myocardial Infarction,” Medicina 60 (2024): 149, 10.3390/medicina60010149. 38256409 PMC10819680

[32] A. Caporali , M. Anwar , Y. Devaux , et al., “Non‐coding RNAs as Therapeutic Targets and Biomarkers in Ischaemic Heart Disease,” Nature Reviews Cardiology 21 (2024): 556–573, 10.1038/s41569-024-01001-5. 38499868

[33] R. Eshraghi , M. Rafiei , Z. Hadian Jazi , D. Shafie , A. Raisi , and H. Mirzaei , “MicroRNA‐155 and Exosomal microRNA‐155: Small Pieces in the Cardiovascular Diseases Puzzle,” Pathology—Research and Practice 257 (2024): 155274, 10.1016/j.prp.2024.155274. 38626659

[34] Z. Mourelatos , J. Dostie , S. Paushkin , et al., “miRNPs: a Novel Class of Ribonucleoproteins Containing Numerous microRNAs,” Genes & Development 16 (2002): 720–728, 10.1101/gad.974702. 11914277 PMC155365

[35] K. Fitzgerald , S. White , A. Borodovsky , et al., “A Highly Durable RNAi Therapeutic Inhibitor of PCSK9,” New England Journal of Medicine 376 (2017): 41–51, 10.1056/NEJMoa1609243. 27959715 PMC5778873

[36] D. Adams , A. Gonzalez‐Duarte , W. D. O'Riordan , et al., “Patisiran, an RNAi Therapeutic, for Hereditary Transthyretin Amyloidosis,” New England Journal of Medicine 379 (2018): 11, 10.1056/NEJMoa1716153. 29972753

[37] M. D. Pérez‐Carrión , I. Posadas , and V. Ceña , “Nanoparticles and siRNA: a New Era in Therapeutics?,” Pharmacological Research 201 (2024): 107102, 10.1016/j.phrs.2024.107102. 38331236

[38] D. Adams , I. L. Tournev , M. S. Taylor , et al., “Efficacy and Safety of vutrisiran for Patients with Hereditary Transthyretin‐mediated Amyloidosis with Polyneuropathy: a Randomized Clinical Trial,” Amyloid 30 (2023): 18–26, 10.1080/13506129.2022.2091985. 35875890

[39] E. Sardh , P. Harper , M. Balwani , et al., “Phase 1 Trial of an RNA Interference Therapy for Acute Intermittent Porphyria,” New England Journal of Medicine 380 (2019): 549–558, 10.1056/NEJMoa1807838. 30726693

[40] Y. Frishberg , G. Deschênes , J. W. Groothoff , et al., “Phase 1/2 Study of Lumasiran for Treatment of Primary Hyperoxaluria Type 1 A Placebo‐Controlled Randomized Clinical Trial,” Clinical Journal of the American Society of Nephrology 16 (2021): 1025–1036, 10.2215/CJN.14730920. 33985991 PMC8425611

[41] J. C. Lieske , G. Ariceta , J. W. Groothoff , et al., “PHYOX3: Nedosiran Long‐Term Safety and Efficacy in Patients with Primary Hyperoxaluria Type 1,” Kidney International Reports 10 (2025): 1993–2002, 10.1016/j.ekir.2025.03.031. 40630298 PMC12231033

[42] S. Sahoo , T. Kariya , and K. Ishikawa , “Targeted Delivery of Therapeutic Agents to the Heart,” Nature Reviews Cardiology 18 (2021): 389–399, 10.1038/s41569-020-00499-9. 33500578 PMC8140998

[43] G. Saenz‐Pipaon and D. A. Dichek , “Targeting and Delivery of microRNA‐targeting Antisense Oligonucleotides in Cardiovascular Diseases,” Atherosclerosis 374 (2023): 44–54, 10.1016/j.atherosclerosis.2022.12.003. 36577600 PMC10277317

[44] B. Laggerbauer and S. Engelhardt , “MicroRNAs as Therapeutic Targets in Cardiovascular Disease,” Journal of Clinical Investigation 132 (2022): 159179, 10.1172/JCI159179. PMC9151703 35642640

[45] P. Gil‐Cabrerizo , T. Simon‐Yarza , E. Garbayo , and M. J. Blanco‐Prieto , “Navigating the Landscape of RNA Delivery Systems in Cardiovascular Disease Therapeutics,” Advanced Drug Delivery Reviews 208 (2024): 115302, 10.1016/j.addr.2024.115302. 38574952

[46] W. H. Organization , 2024.

[47] M. P. Ferreira , V. Balasubramanian , J. Hirvonen , H. Ruskoaho , and H. A. Santos , “Advanced Nanomedicines for the Treatment and Diagnosis of Myocardial Infarction and Heart Failure,” Current Drug Targets 16 (2015): 1682–1697, 10.2174/1389450115999141030143923. 25146697

[48] D. Adhikary , S. Barman , R. Ranjan , and H. Stone , “A Systematic Review of Major Cardiovascular Risk Factors: A Growing Global Health Concern,” Cureus 14 (2022): 30119, 10.7759/cureus.30119. PMC9644238 36381818

[49] B. Sjögren , C. Bigert , and P. Gustavsson , Handbook on the Toxicology of Metals, (Fourth Edition), (Eds: G. F. Nordberg , B. A. Fowler , and M. Nordberg ), (Academic Press, 2015).

[50] S. E. Engelen , A. J. B. Robinson , Y.‐X. Zurke , and C. Monaco , “Therapeutic Strategies Targeting Inflammation and Immunity in Atherosclerosis: How to Proceed?,” Nature Reviews Cardiology 19 (2022): 522–542, 10.1038/s41569-021-00668-4. 35102320 PMC8802279

[51] M. Naghavi , P. Libby , E. Falk , et al., “From Vulnerable Plaque to Vulnerable Patient,” Circulation 108 (2003): 1664–1672, 10.1161/01.CIR.0000087480.94275.97. 14530185

[52] S. S. Virani , L. K. Newby , S. V. Arnold , et al., “2023 AHA/ACC/ACCP/ASPC/NLA/PCNA Guideline for the Management of Patients with Chronic Coronary Disease: a Report of the American Heart Association/American College of Cardiology Joint Committee on Clinical Practice Guidelines,” Circulation 148 (2023): 9, 10.1161/CIR.0000000000001168. 37471501

[53] D. A. Ojha N , (2024).

[54] X. He , T. Du , T. Long , X. Liao , Y. Dong , and Z.‐P. Huang , “Signaling Cascades in the Failing Heart and Emerging Therapeutic Strategies,” Signal Transduction and Targeted Therapy 7 (2022): 134, 10.1038/s41392-022-00972-6. 35461308 PMC9035186

[55] M. P. A. Ferreira , S. Ranjan , A. M. R. Correia , et al., “In Vitro and in Vivo Assessment of Heart‐homing Porous Silicon Nanoparticles,” Biomaterials 94 (2016): 93–104, 10.1016/j.biomaterials.2016.03.046. 27107168

[56] M. P. A. Ferreira , S. Ranjan , S. Kinnunen , et al., “Drug‐Loaded Multifunctional Nanoparticles Targeted to the Endocardial Layer of the Injured Heart Modulate Hypertrophic Signaling,” Small 13 (2017): 1701276, 10.1002/smll.201701276. 28714245

[57] N. G. Frangogiannis , “Regulation of the Inflammatory Response in Cardiac Repair,” Circulation Research 110 (2012): 159–173, 10.1161/CIRCRESAHA.111.243162. 22223212 PMC3690135

[58] A. S. Riching and K. Song , “Cardiac Regeneration: New Insights into the Frontier of Ischemic Heart Failure Therapy,” Frontiers in Bioengineering and Biotechnology 8 (2020): 637538, 10.3389/fbioe.2020.637538. 33585427 PMC7873479

[59] M. P. A. Ferreira , V. Talman , G. Torrieri , et al., “Dual‐Drug Delivery Using Dextran‐Functionalized Nanoparticles Targeting Cardiac Fibroblasts for Cellular Reprogramming,” Advanced Functional Materials 28 (2018): 1705134, 10.1002/adfm.201705134.

[60] G. Torrieri , I. Iqbal , F. Fontana , et al., “Macrophage Hitchhiking Nanoparticles for the Treatment of Myocardial Infarction: an in Vitro and in Vivo Study,” Advanced Functional Materials 33 (2023): 2303658, 10.1002/adfm.202303658.

[61] G. Torrieri , F. Fontana , P. Figueiredo , et al., “Dual‐peptide Functionalized Acetalated Dextran‐based Nanoparticles for Sequential Targeting of Macrophages during Myocardial Infarction,” Nanoscale 12 (2020): 2350–2358, 10.1039/C9NR09934D. 31930241

[62] Y. Shao , C. Xu , S. Zhu , et al., “One Endothelium‐Targeted Combined Nucleic Acid Delivery System for Myocardial Infarction Therapy,” ACS Nano 18 (2024): 8107–8124, 10.1021/acsnano.3c11661. 38442075

[63] Y. Zhou , Q. Liang , X. Wu , et al., “siRNA Delivery against Myocardial Ischemia Reperfusion Injury Mediated by Reversibly Camouflaged Biomimetic Nanocomplexes,” Advanced Materials 35 (2023): 2210691, 10.1002/adma.202210691. 36913720

[64] J. Wang , S. Liu , T. Heallen , and J. F. Martin , “The Hippo Pathway in the Heart: Pivotal Roles in Development, Disease, and Regeneration,” Nature Reviews Cardiology 15 (2018): 672–684, 10.1038/s41569-018-0063-3. 30111784

[65] J. P. Leach , T. Heallen , M. Zhang , et al., “Hippo Pathway Deficiency Reverses Systolic Heart Failure after Infarction,” Nature 550 (2017): 260–264, 10.1038/nature24045. 28976966 PMC5729743

[66] Q. Zhang , L. Wang , S. Wang , et al., “Signaling Pathways and Targeted Therapy for Myocardial Infarction,” Signal Transduction and Targeted Therapy 7 (2022): 78, 10.1038/s41392-022-00925-z. 35273164 PMC8913803

[67] W.‐F. Cai , L. Wang , G.‐S. Liu , P. Zhu , C. Paul , and Y. Wang , “Manipulating the Hippo‐Yap Signal Cascade in Stem Cells for Heart Regeneration,” Annals of Palliative Medicine 5 (2016): 125–134, 10.21037/apm.2016.03.03. 27121740

[68] A. Ghigo , M. Laffargue , M. Li , and E. Hirsch , “PI3K and Calcium Signaling in Cardiovascular Disease,” Circulation Research 121 (2017): 282–292, 10.1161/CIRCRESAHA.117.310183. 28729453

[69] T. C. T. M. van der Pouw Kraan , F. J. P. Bernink , C. Yildirim , et al., “Systemic Toll‐Like Receptor and Interleukin‐18 Pathway Activation in Patients with Acute ST Elevation Myocardial Infarction,” Journal of Molecular and Cellular Cardiology 67 (2014): 94–102, 10.1016/j.yjmcc.2013.12.021. 24389343

[70] J. Zoldan , T. P. Kraehenbuehl , A. K. R. Lytton‐Jean , R. S. Langer , and D. G. Anderson , “Tissue Engineering for Stem Cell Mediated Regenerative Medicine,” in Human Stem Cell Technology and Biology: A Research Guide and Laboratory Manual (Wiley Press, 2010), 10.1002/9780470889909.ch31.

[71] J. Zoldan , A. K. R. Lytton‐Jean , E. D. Karagiannis , et al., “Directing human Embryonic Stem Cell Differentiation by Non‐viral Delivery of siRNA in 3D Culture,” Biomaterials 32 (2011): 7793–7800, 10.1016/j.biomaterials.2011.06.057. 21835461 PMC3313656

[72] J. Palasubramaniam , X. Wang , and K. Peter , “Myocardial Infarction—From Atherosclerosis to Thrombosis: Uncovering New Diagnostic and Therapeutic Approaches,” Arteriosclerosis, Thrombosis, and Vascular Biology 39 (2019): 176, 10.1161/ATVBAHA.119.312578. 31339782

[73] O. Soehnlein and P. Libby , “Targeting Inflammation in Atherosclerosis — From Experimental Insights to the Clinic,” Nature Reviews Drug Discovery 20 (2021): 589–610, 10.1038/s41573-021-00198-1. 33976384 PMC8112476

[74] A. C. Aprotosoaie , A. D. Costache , and I. I. Costache , “Therapeutic Strategies and Chemoprevention of Atherosclerosis: What Do We Know and Where Do We Go?,” Pharmaceutics 14 (2022): 722, 10.3390/pharmaceutics14040722. 35456556 PMC9025701

[75] Y. N. Lamb , “Inclisiran: First Approval,” Drugs 81 (2021): 389–395, 10.1007/s40265-021-01473-6. 33620677 PMC7900795

[76] K. K. Ray , R. P. T. Troquay , F. L. J. Visseren , et al., “Long‐term Efficacy and Safety of inclisiran in Patients with High Cardiovascular Risk and Elevated LDL Cholesterol (ORION‐3): Results from the 4‐year Open‐label Extension of the ORION‐1 Trial,” The Lancet Diabetes & Endocrinology 11 (2023): 109–119, 10.1016/S2213-8587(22)00353-9. 36620965

[77] S. Kettunen , A. K. Ruotsalainen , and S. Ylä‐Herttuala , “RNA Interference‐based Therapies for the Control of Atherosclerosis Risk Factors,” Current Opinion in Cardiology 37 (2022): 364–371, 10.1097/HCO.0000000000000972. 35731681

[78] W. Tao , A. Yurdagul , N. Kong , et al., “siRNA Nanoparticles Targeting CaMKIIγ in Lesional Macrophages Improve Atherosclerotic Plaque Stability in Mice,” Science Translational Medicine 12 (2020): aay1063, 10.1126/scitranslmed.aay1063. PMC7476570 32718990

[79] Y. Zhao , H. Gao , J. He , et al., “Co‐delivery of LOX‐1 siRNA and Statin to Endothelial Cells and Macrophages in the Atherosclerotic Lesions by a Dual‐targeting Core‐shell Nanoplatform: a Dual Cell Therapy to Regress Plaques,” Journal of Controlled Release 283 (2018): 241–260, 10.1016/j.jconrel.2018.05.041. 29885417

[80] T. Jiang , L. Xu , M. Zhao , et al., “Dual Targeted Delivery of Statins and Nucleic Acids by Chitosan‐based Nanoparticles for Enhanced Antiatherosclerotic Efficacy,” Biomaterials 280 (2022): 121324, 10.1016/j.biomaterials.2021.121324. 34933253

[81] M. Gao , M. Tang , W. Ho , et al., “Modulating Plaque Inflammation via Targeted mRNA Nanoparticles for the Treatment of Atherosclerosis,” ACS Nano 17 (2023): 17721–17739, 10.1021/acsnano.3c00958. 37669404

[82] K. M. Owsiany , G. F. Alencar , and G. K. Owens , “Revealing the Origins of Foam Cells in Atherosclerotic Lesions,” Arteriosclerosis, Thrombosis, and Vascular Biology 39 (2019): 836–838, 10.1161/ATVBAHA.119.312557. 31017823 PMC6482855

[83] A. V. Poznyak , N. G. Nikiforov , A. V. Starodubova , T. V. Popkova , and A. N. Orekhov , “Macrophages and Foam Cells: Brief Overview of Their Role, Linkage, and Targeting Potential in Atherosclerosis,” Biomedicines 9 (2021): 1221, 10.3390/biomedicines9091221. 34572406 PMC8468383

[84] K. Kim , D. Shim , J. S. Lee , et al., “Transcriptome Analysis Reveals Nonfoamy Rather than Foamy Plaque Macrophages Are Proinflammatory in Atherosclerotic Murine Models,” Circulation Research 123 (2018): 1127–1142, 10.1161/CIRCRESAHA.118.312804. 30359200 PMC6945121

[85] A. Javadifar , S. Rastgoo , M. Banach , T. Jamialahmadi , T. P. Johnston , and A. Sahebkar , “Foam Cells as Therapeutic Targets in Atherosclerosis with a Focus on the Regulatory Roles of Non‐Coding RNAs,” International Journal of Molecular Sciences 22 (2021): 2529, 10.3390/ijms22052529. 33802600 PMC7961492

[86] D. M. Fernandez and C. Giannarelli , “Immune Cell Profiling in Atherosclerosis: Role in Research and Precision Medicine,” Nature Reviews Cardiology 19 (2022): 43–58, 10.1038/s41569-021-00589-2. 34267377 PMC8280607

[87] M. Piollet , F. Porsch , G. Rizzo , et al., “TREM2 protects from Atherosclerosis by Limiting Necrotic Core Formation,” Nature Cardiovascular Research 3 (2024): 269–282, 10.1038/s44161-024-00429-9. PMC7616136 38974464

[88] Y. Gui , H. Zheng , and R. Y. Cao , “Foam Cells in Atherosclerosis: Novel Insights into Its Origins, Consequences, and Molecular Mechanisms,” Frontiers in Cardiovascular Medicine 9 (2022): 845942, 10.3389/fcvm.2022.845942. 35498045 PMC9043520

[89] Y. Zhao , Z. He , H. Gao , et al., “Fine Tuning of Core–Shell Structure of Hyaluronic Acid/Cell‐Penetrating Peptides/siRNA Nanoparticles for Enhanced Gene Delivery to Macrophages in Antiatherosclerotic Therapy,” Biomacromolecules 19 (2018): 2944–2956, 10.1021/acs.biomac.8b00501. 29641895

[90] B. Pelaz , C. Alexiou , R. A. Alvarez‐Puebla , et al., “Diverse Applications of Nanomedicine,” ACS Nano 11 (2017): 2313–2381, 10.1021/acsnano.6b06040. 28290206 PMC5371978

[91] P. M. Martins , A. C. Lima , S. Ribeiro , S. Lanceros‐Mendez , and P. Martins , “Magnetic Nanoparticles for Biomedical Applications: from the Soul of the Earth to the Deep History of Ourselves,” ACS Applied Bio Materials 4 (2021): 5839–5870, 10.1021/acsabm.1c00440. 35006927

[92] M. A. Tölli , M. P. A. Ferreira , S. M. Kinnunen , et al., “In Vivo Biocompatibility of Porous Silicon Biomaterials for Drug Delivery to the Heart,” Biomaterials 35 (2014): 8394–8405, 10.1016/j.biomaterials.2014.05.078. 24985734

[93] R. S. Soumya and K. G. Raghu , “Recent Advances on Nanoparticle‐based Therapies for Cardiovascular Diseases,” Journal of Cardiology 81 (2023): 10–18, 10.1016/j.jjcc.2022.02.009. 35210166

[94] Q. Hu , Z. Fang , J. Ge , and H. Li , “Nanotechnology for Cardiovascular Diseases,” Innovation (Camb) 3 (2022): 100214, 10.1016/j.xinn.2022.100214. 35243468 PMC8866095

[95] Z. Liu , S. Wang , C. Tapeinos , et al., “Non‐viral Nanoparticles for RNA Interference: Principles of Design and Practical Guidelines,” Advanced Drug Delivery Reviews 174 (2021): 576–612, 10.1016/j.addr.2021.05.018. 34019958

[96] B. Hu , L. Zhong , Y. Weng , et al., “Therapeutic siRNA: State of the Art,” Signal Transduction and Targeted Therapy 5 (2020): 101, 10.1038/s41392-020-0207-x. 32561705 PMC7305320

[97] P. Ranasinghe , M. L. Addison , J. W. Dear , and D. J. Webb , “Small Interfering RNA: Discovery, Pharmacology and Clinical Development—An Introductory Review,” British Journal of Pharmacology 180 (2023): 2697–2720, 10.1111/bph.15972. 36250252

[98] I. V. Chernikov , U. A. Ponomareva , and E. L. Chernolovskaya , “Structural Modifications of siRNA Improve Its Performance in Vivo,” International Journal of Molecular Sciences 24 (2023): 956, 10.3390/ijms24020956. 36674473 PMC9862127

[99] M. R. Hassler , A. A. Turanov , J. F. Alterman , et al., “Comparison of Partially and Fully Chemically‐modified siRNA in Conjugate‐mediated Delivery in Vivo,” Nucleic Acids Research 46 (2018): 2185–2196, 10.1093/nar/gky037. 29432571 PMC5861422

[100] R. A. Haraszti , L. Roux , A. H. Coles , et al., “5΄‐Vinylphosphonate Improves Tissue Accumulation and Efficacy of Conjugated siRNAs in Vivo,” Nucleic Acids Research 45 (2017): 7581–7592, 10.1093/nar/gkx507. 28591791 PMC5570069

[101] C. Wolfrum , S. Shi , K. N. Jayaprakash , et al., “Mechanisms and Optimization of in Vivo Delivery of Lipophilic siRNAs,” Nature Biotechnology 25 (2007): 1149–1157, 10.1038/nbt1339. 17873866

[102] Y. Dong , D. J. Siegwart , and D. G. Anderson , “Strategies, Design, and Chemistry in siRNA Delivery Systems,” Advanced Drug Delivery Reviews 144 (2019): 133–147, 10.1016/j.addr.2019.05.004. 31102606 PMC6745264

[103] W. A. Velema and E. T. Kool , “The Chemistry and Applications of RNA 2′‐OH Acylation,” Nature Reviews Chemistry 4 (2020): 22–37, 10.1038/s41570-019-0147-6. PMC7513686 32984545

[104] J. Witten , Y. Hu , R. Langer , and D. G. Anderson , “Recent Advances in Nanoparticulate RNA Delivery Systems,” Proceedings of the National Academy of Sciences 121 (2024): 2307798120, 10.1073/pnas.2307798120. PMC10945842 38437569

[105] B. Kim , J.‐H. Park , and M. J. Sailor , “Rekindling RNAi Therapy: Materials Design Requirements for in Vivo siRNA Delivery,” Advanced Materials 31 (2019): 1903637, 10.1002/adma.201903637. PMC6891135 31566258

[106] M. Lavertu , S. Méthot , N. Tran‐Khanh , and M. D. Buschmann , “High Efficiency Gene Transfer Using Chitosan/DNA Nanoparticles with Specific Combinations of Molecular Weight and Degree of Deacetylation,” Biomaterials 27 (2006): 4815–4824, 10.1016/j.biomaterials.2006.04.029. 16725196

[107] D. J. Gary , J. Min , Y. Kim , K. Park , and Y. Y. Won , “The Effect of N/ P Ratio on the in Vitro and in Vivo Interaction Properties of PEG Ylated Poly[2‐(dimethylamino)ethyl methacrylate]‐ B Ased si RNA Complexes,” Macromolecular Bioscience 13 (2013): 1059–1071, 10.1002/mabi.201300046. 23828845 PMC4121118

[108] B. Ballarín‐González , F. Dagnaes‐Hansen , R. A. Fenton , et al., “Protection and Systemic Translocation of siRNA Following Oral Administration of Chitosan/siRNA Nanoparticles,” Molecular Therapy—Nucleic Acids 2, no. 2 (2013): 76, 10.1038/mtna.2013.2. PMC4098703 23462963

[109] F. Wang , J. D. Pang , L. L. Huang , et al., “Nanoscale Polysaccharide Derivative as an AEG‐1 siRNA Carrier for Effective Osteosarcoma Therapy,” International Journal of Nanomedicine 13 (2018): 857–875, 10.2147/IJN.S147747. 29467575 PMC5811182

[110] K. Raemdonck , T. F. Martens , K. Braeckmans , J. Demeester , and S. C. De Smedt , “Polysaccharide‐based Nucleic Acid Nanoformulations,” Advanced Drug Delivery Reviews 65 (2013): 1123–1147, 10.1016/j.addr.2013.05.002. 23680381

[111] S. Mizrahy and D. Peer , “Polysaccharides as Building Blocks for Nanotherapeutics,” Chemical Society Reviews 41 (2012): 2623–2640, 10.1039/C1CS15239D. 22085917

[112] M. Paidikondala , V. K. Rangasami , G. N. Nawale , et al., “An Unexpected Role of Hyaluronic Acid in Trafficking siRNA across the Cellular Barrier: the First Biomimetic, Anionic, Non‐Viral Transfection Method,” Angewandte Chemie International Edition 58 (2019): 2815–2819, 10.1002/anie.201900099. 30644615

[113] W. Poon , B. R. Kingston , B. Ouyang , W. Ngo , and W. C. W. Chan , “A Framework for Designing Delivery Systems,” Nature Nanotechnology 15 (2020): 819–829, 10.1038/s41565-020-0759-5. 32895522

[114] P. Joyce , C. J. Allen , M. J. Alonso , et al., “A Translational Framework to DELIVER Nanomedicines to the Clinic,” Nature Nanotechnology 19 (2024): 1597–1611. 10.1038/s41565-024-01754-7 39242807

[115] S. J. Jenkins and D. A. Hume , “Homeostasis in the Mononuclear Phagocyte System,” Trends in Immunology 35 (2014): 358–367, 10.1016/j.it.2014.06.006. 25047416

[116] E. Blanco , H. Shen , and M. Ferrari , “Principles of Nanoparticle Design for Overcoming Biological Barriers to Drug Delivery,” Nature Biotechnology 33 (2015): 941–951, 10.1038/nbt.3330. PMC4978509 26348965

[117] A. E. Nel , L. Mädler , D. Velegol , et al., “Understanding Biophysicochemical Interactions at the Nano–bio Interface,” Nature Materials 8 (2009): 543–557, 10.1038/nmat2442. 19525947

[118] T. Lima , K. Bernfur , M. Vilanova , and T. Cedervall , “Understanding the Lipid and Protein Corona Formation on Different Sized Polymeric Nanoparticles,” Scientific Reports 10 (2020): 1129, 10.1038/s41598-020-57943-6. 31980686 PMC6981174

[119] W. Wang , Z. Huang , Y. Li , et al., “Impact of Particle Size and pH on Protein Corona Formation of Solid Lipid Nanoparticles: a Proof‐of‐concept Study,” Acta Pharmaceutica Sinica B 11 (2021): 1030–1046, 10.1016/j.apsb.2020.10.023. 33996415 PMC8105779

[120] R. García‐Álvarez , M. Hadjidemetriou , A. Sánchez‐Iglesias , L. M. Liz‐Marzán , and K. Kostarelos , “In Vivo Formation of Protein Corona on Gold Nanoparticles. The Effect of Their Size and Shape,” Nanoscale 10 (2018): 1256–1264, 10.1039/C7NR08322J. 29292433

[121] M. Dolci , Y. Wang , S. W. Nooteboom , et al., “Real‐Time Optical Tracking of Protein Corona Formation on Single Nanoparticles in Serum,” ACS Nano 17 (2023): 20167–20178, 10.1021/acsnano.3c05872. 37802067 PMC10604089

[122] D. Chen , S. Ganesh , W. Wang , and M. Amiji , “The Role of Surface Chemistry in Serum Protein Corona‐mediated Cellular Delivery and Gene Silencing with Lipid Nanoparticles,” Nanoscale 11 (2019): 8760–8775, 10.1039/C8NR09855G. 30793730

[123] B. D. Johnston , W. G. Kreyling , C. Pfeiffer , et al., “Colloidal Stability and Surface Chemistry Are Key Factors for the Composition of the Protein Corona of Inorganic Gold Nanoparticles,” Advanced Functional Materials 27 (2017): 1701956, 10.1002/adfm.201701956.

[124] V. Francia , K. Yang , S. Deville , C. Reker‐Smit , I. Nelissen , and A. Salvati , “Corona Composition Can Affect the Mechanisms Cells Use to Internalize Nanoparticles,” ACS Nano 13 (2019): 11107, 10.1021/acsnano.9b03824. 31525954 PMC6812477

[125] F. Giulimondi , L. Digiacomo , D. Pozzi , et al., “Interplay of Protein Corona and Immune Cells Controls Blood Residency of Liposomes,” Nature Communications 10 (2019): 3686, 10.1038/s41467-019-11642-7. PMC6695391 31417080

[126] C. M. Hu , R. H. Fang , K. C. Wang , et al., “Nanoparticle Biointerfacing by Platelet Membrane Cloaking,” Nature 526 (2015): 118–121, 10.1038/nature15373. 26374997 PMC4871317

[127] M. D. McSweeney , T. Wessler , L. S. L. Price , et al., “A Minimal Physiologically Based Pharmacokinetic Model That Predicts Anti‐PEG IgG‐mediated Clearance of PEGylated Drugs in human and Mouse,” Journal of Controlled Release 284 (2018): 171–178, 10.1016/j.jconrel.2018.06.002. 29879519 PMC6087483

[128] Y.‐H. Cheng , C. He , J. E. Riviere , N. A. Monteiro‐Riviere , and Z. Lin , “Meta‐Analysis of Nanoparticle Delivery to Tumors Using a Physiologically Based Pharmacokinetic Modeling and Simulation Approach,” ACS Nano 14 (2020): 3075–3095, 10.1021/acsnano.9b08142. 32078303 PMC7098057

[129] L. Scheetz , K. S. Park , Q. Li , et al., “Engineering Patient‐specific Cancer Immunotherapies,” Nature Biomedical Engineering 3 (2019): 768–782, 10.1038/s41551-019-0436-x. PMC6783331 31406259

[130] J. J. Rennick , A. P. R. Johnston , and R. G. Parton , “Key Principles and Methods for Studying the Endocytosis of Biological and Nanoparticle Therapeutics,” Nature Nanotechnology 16 (2021): 266–276, 10.1038/s41565-021-00858-8. 33712737

[131] I. M. S. Degors , C. Wang , Z. U. Rehman , and I. S. Zuhorn , “Carriers Break Barriers in Drug Delivery: Endocytosis and Endosomal Escape of Gene Delivery Vectors,” Accounts of Chemical Research 52 (2019): 1750–1760, 10.1021/acs.accounts.9b00177. 31243966 PMC6639780

[132] M. Jovic , M. Sharma , J. Rahajeng , and S. Caplan , “The Early Endosome: a Busy Sorting Station for Proteins at the Crossroads,” Histology and Histopathology 25 (2010): 99–112, 10.14670/hh-25.99. 19924646 PMC2810677

[133] S. Patel , J. Kim , M. Herrera , A. Mukherjee , A. V. Kabanov , and G. Sahay , “Brief Update on Endocytosis of Nanomedicines,” Advanced Drug Delivery Reviews 144 (2019): 90–111, 10.1016/j.addr.2019.08.004. 31419450 PMC6986687

[134] J. Gilleron , W. Querbes , A. Zeigerer , et al., “Image‐based Analysis of Lipid Nanoparticle–mediated siRNA Delivery, Intracellular Trafficking and Endosomal Escape,” Nature Biotechnology 31 (2013): 638–646, 10.1038/nbt.2612. 23792630

[135] A. Wittrup , A. Ai , X. Liu , et al., “Visualizing Lipid‐formulated siRNA Release from Endosomes and Target Gene Knockdown,” Nature Biotechnology 33 (2015): 870, 10.1038/nbt.3298. PMC4663660 26192320

[136] J. Huotari and A. Helenius , “Endosome Maturation,” The EMBO Journal 30 (2011): 3481–3500, 10.1038/emboj.2011.286. 21878991 PMC3181477

[137] H. Du Rietz , H. Hedlund , S. Wilhelmson , P. Nordenfelt , and A. Wittrup , “Imaging Small Molecule‐induced Endosomal Escape of siRNA,” Nature Communications 11 (2020): 1809, 10.1038/s41467-020-15300-1. PMC7156650 32286269

[138] H. Gao , S. Wang , Q. Long , et al., “Rational Design of a Polysaccharide‐based Viral Mimicry Nanocomplex for Potent Gene Silencing in Inflammatory Tissues,” Journal of Controlled Release 357 (2023): 120–132, 10.1016/j.jconrel.2023.03.037. 36963635

[139] J.‐H. Wang , D. J. Gessler , W. Zhan , T. L. Gallagher , and G. Gao , “Adeno‐associated Virus as a Delivery Vector for Gene Therapy of human Diseases,” Signal Transduction and Targeted Therapy 9 (2024): 78, 10.1038/s41392-024-01780-w. 38565561 PMC10987683

[140] N. Hammoudi , K. Ishikawa , and R. J. Hajjar , “Adeno‐associated Virus‐mediated Gene Therapy in Cardiovascular Disease,” Current Opinion in Cardiology 30 (2015): 228–234, 10.1097/HCO.0000000000000159. 25783685 PMC4417622

[141] J. Gao , Y. Guo , Y. Chen , J. Zhou , Y. Liu , and P. Su , “Adeno‐associated Virus 9‐mediated RNA Interference Targeting SOCS3 Alleviates Diastolic Heart Failure in Rats,” Gene 697 (2019): 11–18, 10.1016/j.gene.2019.01.044. 30763670

[142] S. Pillay , W. Zou , F. Cheng , et al., “Adeno‐associated Virus (AAV) Serotypes Have Distinctive Interactions with Domains of the Cellular AAV Receptor,” Journal of Virology 91 (2017): 10–1128, 10.1128/jvi.00391-17. PMC5571256 28679762

[143] C. Neuber , O. J. Müller , F. C. Hansen , et al., “Paradoxical Effects on Force Generation after Efficient β1‐Adrenoceptor Knockdown in Reconstituted Heart Tissue,” The Journal of Pharmacology and Experimental Therapeutics 349 (2014): 39–46, 10.1124/jpet.113.210898. 24431469

[144] M. J. Wright , L. M. Wightman , C. Lilley , et al., “In Vivo Myocardial Gene Transfer: Optimization, Evaluation and Direct Comparison of Gene Transfer Vectors,” Basic Research in Cardiology 96 (2001): 227–236, 10.1007/s003950170053. 11403416

[145] J. R. Mendell , A. M. Connolly , K. J. Lehman , et al., “Testing Preexisting Antibodies Prior to AAV Gene Transfer Therapy: Rationale, Lessons and Future Considerations,” Molecular Therapy—Methods & Clinical Development 25 (2022): 74–83, 10.1016/j.omtm.2022.02.011. 35356756 PMC8933338

[146] Y. Kim , A. P. Landstrom , S. H. Shah , J. C. Wu , and C. E. Seidman , “Gene Therapy in Cardiovascular Disease: Recent Advances and Future Directions in Science: a Science Advisory from the American Heart Association,” Circulation 150 (2024): 471, 10.1161/CIR.0000000000001296. 39523949

[147] E. J. Loeb , S. A. Fergione , V. Yudistyra , et al., “Complete Neutralizing Antibody Evasion by Serodivergent Non‐mammalian AAVs Enables Gene Therapy Redosing,” Cell Reports Medicine 6 (2025): 102475, 10.1016/j.xcrm.2025.102475. 41308640 PMC12765845

[148] M. Muhuri , Y. Maeda , H. Ma , et al., “Novel Combinatorial MicroRNA‐Binding Sites in AAV Vectors Synergistically Diminish Antigen Presentation and Transgene Immunity for Efficient and Stable Transduction,” Journal of Clinical Investigation 131 (2021): 674242, 10.3389/fimmu.2021.674242. PMC8113644 33995418

[149] G. S. Tomassy , W. Fan , S. Cao , et al., “Development of an AAV‐delivered microRNA Gene Therapy for Myotonic Dystrophy Type 1,” Molecular Therapy 33 (2025): 6350–6365, 10.1016/j.ymthe.2025.08.050. 40903903 PMC12703144

[150] L. Switala , L. Di , H. Gao , et al., “Engineered Nanoparticles Promote Cardiac Tropism of AAV Vectors,” Journal of Nanobiotechnology 22 (2024): 223, 10.1186/s12951-024-02485-6. 38702815 PMC11067271

[151] K. Luo , Z. Zhang , S. Yao , Y. Wang , M. Amiji , and K. C. Anderson , “Advances in RNA Therapeutics and Its Delivery Strategies against Multiple Myeloma,” Journal of Controlled Release 385 (2025): 114048, 10.1016/j.jconrel.2025.114048. 40691968

[152] Z. Zhang , S. Yao , Y. Hu , X. Zhao , and R. J. Lee , “Application of Lipid‐based Nanoparticles in Cancer Immunotherapy,” Frontiers in Immunology 13 (2022): 967505, 10.3389/fimmu.2022.967505. 36003395 PMC9393708

[153] M. C. I. Labonia , M. Estapé Senti , P. H. van der Kraak , et al., “Cardiac Delivery of Modified mRNA Using Lipid Nanoparticles: Cellular Targets and Biodistribution after Intramyocardial Administration,” Journal of Controlled Release 369 (2024): 734–745, 10.1016/j.jconrel.2024.04.018. 38604385

[154] Y. Zeng , M. E. Senti , M. C. I. Labonia , et al., “Fusogenic Coiled‐Coil Peptides Enhance Lipid Nanoparticle‐Mediated mRNA Delivery Upon Intramyocardial Administration,” ACS Nano 17 (2023): 23466–23477, 10.1021/acsnano.3c05341. 37982378 PMC10722601

[155] S. A. Dilliard and D. J. Siegwart , “Passive, Active and Endogenous Organ‐targeted Lipid and Polymer Nanoparticles for Delivery of Genetic Drugs,” Nature Reviews Materials 8 (2023): 282–300, 10.1038/s41578-022-00529-7. 36691401 PMC9850348

[156] C. Vergallo , G. Torrieri , R. Provenzani , et al., “Design, Synthesis and Characterization of a PEGylated Stanozolol for Potential Therapeutic Applications,” International Journal of Pharmaceutics 573 (2020): 118826, 10.1016/j.ijpharm.2019.118826. 31715352

[157] J. A. Kulkarni , P. R. Cullis , and R. van der Meel , “Lipid Nanoparticles Enabling Gene Therapies: from Concepts to Clinical Utility,” Nucleic Acid Therapeutics 28 (2018): 146–157, 10.1089/nat.2018.0721. 29683383

[158] A. S. Piotrowski‐Daspit , A. C. Kauffman , L. G. Bracaglia , and W. M. Saltzman , “Polymeric Vehicles for Nucleic Acid Delivery,” Advanced Drug Delivery Reviews 156 (2020): 119–132, 10.1016/j.addr.2020.06.014. 32585159 PMC7736472

[159] X. Cai , R. Dou , C. Guo , et al., “Cationic Polymers as Transfection Reagents for Nucleic Acid Delivery,” Pharmaceutics 15 (2023): 1502, 10.3390/pharmaceutics15051502. 37242744 PMC10223806

[160] J. Casper , S. H. Schenk , E. Parhizkar , P. Detampel , A. Dehshahri , and J. Huwyler , “Polyethylenimine (PEI) in Gene Therapy: Current Status and Clinical Applications,” Journal of Controlled Release 362 (2023): 667–691, 10.1016/j.jconrel.2023.09.001. 37666302

[161] A. M. Weiss , M. A. Lopez, II , B. W. Rawe , et al., “Understanding How Cationic Polymers′ Properties Inform Toxic or Immunogenic Responses via Parametric Analysis,” Macromolecules 56 (2023): 7286–7299, 10.1021/acs.macromol.3c01223. 37781211 PMC10537447

[162] F. Wang , L. Gao , L.‐Y. Meng , J.‐M. Xie , J.‐W. Xiong , and Y. Luo , “A Neutralized Noncharged Polyethylenimine‐Based System for Efficient Delivery of siRNA into Heart without Toxicity,” ACS Applied Materials & Interfaces 8 (2016): 33529–33538, 10.1021/acsami.6b13295. 27960377

[163] K. Paunovska , D. Loughrey , and J. E. Dahlman , “Drug Delivery Systems for RNA Therapeutics,” Nature Reviews Genetics 23 (2022): 265–280, 10.1038/s41576-021-00439-4. PMC8724758 34983972

[164] M. Krohn‐Grimberghe , M. J. Mitchell , M. J. Schloss , et al., “Nanoparticle‐encapsulated siRNAs for Gene Silencing in the Haematopoietic Stem‐cell Niche,” Nature Biomedical Engineering 4 (2020): 1076–1089, 10.1038/s41551-020-00623-7. PMC7655681 33020600

[165] P. Dosta , C. Demos , V. Ramos , et al., “Delivery of siRNA to Endothelial Cells in Vivo Using Lysine/Histidine Oligopeptide‐Modified Poly(β‐amino ester) Nanoparticles,” Cardiovascular Engineering and Technology 12 (2021): 114–125, 10.1007/s13239-021-00518-x. 33474643 PMC8536891

[166] M. Hou , X. Wu , Z. Zhao , Q. Deng , Y. Chen , and L. Yin , “Endothelial Cell‐targeting, ROS‐ultrasensitive Drug/siRNA co‐delivery Nanocomplexes Mitigate Early‐stage Neutrophil Recruitment for the Anti‐inflammatory Treatment of Myocardial Ischemia Reperfusion Injury,” Acta Biomaterialia 143 (2022): 344–355, 10.1016/j.actbio.2022.02.018. 35189380

[167] H. He , J. Wang , P. J. Yannie , W. J. Korzun , H. Yang , and S. Ghosh , “Nanoparticle‐based “Two‐pronged” Approach to Regress Atherosclerosis by Simultaneous Modulation of Cholesterol Influx and Efflux,” Biomaterials 260 (2020): 120333, 10.1016/j.biomaterials.2020.120333. 32853832 PMC7530139

[168] H. Ni , H. Zhou , X. Liang , et al., “Reactive Oxygen Species‐Responsive Nanoparticle Delivery of Small Interfering Ribonucleic Acid Targeting Olfactory Receptor 2 for Atherosclerosis Theranostics,” ACS Nano 34 (2024): 23599–23614, 10.1021/acsnano.4c07988. 39141682

[169] R. Zhao , J. Guo , Z. Liu , et al., “Zwitterionic Lipid Nanoparticles for Efficient siRNA Delivery and Hypercholesterolemia Therapy with Rational Charge Self‐transformation,” Theranostics 15 (2025): 3693–3712, 10.7150/thno.111685. 40093884 PMC11905131

[170] V. V. Shuvaev , Y. K. Tam , B. W. Lee , et al., “Systemic Delivery of Biotherapeutic RNA to the Myocardium Transiently Modulates Cardiac Contractility in Vivo,” Proceedings of the National Academy of Sciences 122 (2025): 2409266122, 10.1073/pnas.2409266122. PMC12304964 40668829

[171] X. Wang , Y. Zhong , B. Qian , et al., “Self‐assembled Extracellular Matrix‐lipid Nanoparticle Composite for Site‐specific siRNA Delivery to Improve Cardiac Repair Post‐myocardial Infarction,” Materials Today Bio 34 (2025): 102205, 10.1016/j.mtbio.2025.102205. PMC12395518 40893381

[172] I. Serrano‐Sevilla , Á. Artiga , S. G. Mitchell , L. De Matteis , and J. M. de la Fuente , “Natural Polysaccharides for siRNA Delivery: Nanocarriers Based on Chitosan, Hyaluronic Acid, and Their Derivatives,” Molecules (Basel, Switzerland) 24 (2019): 2570, 10.3390/molecules24142570. 31311176 PMC6680562

[173] R. J. Peach , D. Hollenbaugh , I. Stamenkovic , and A. Aruffo , “Identification of Hyaluronic Acid Binding Sites in the Extracellular Domain of CD44,” The Journal of cell biology 122 (1993): 257–264, 10.1083/jcb.122.1.257. 8314845 PMC2119597

[174] M. Raviña , E. Cubillo , D. Olmeda , et al., “Hyaluronic Acid/Chitosan‐g‐Poly(ethylene glycol) Nanoparticles for Gene Therapy: an Application for pDNA and siRNA Delivery,” Pharmaceutical Research 27 (2010): 2544, 10.1007/s11095-010-0263-y. 20857179

[175] K. Park , M. Y. Lee , K. S. Kim , and S. K. Hahn , “Target Specific Tumor Treatment by VEGF siRNA Complexed with Reducible Polyethyleneimine–hyaluronic Acid Conjugate,” Biomaterials 31 (2010): 5258–5265, 10.1016/j.biomaterials.2010.03.018. 20378167

[176] C. Tapeinos , H. Gao , T. Bauleth‐Ramos , and H. A. Santos , “Progress in Stimuli‐Responsive Biomaterials for Treating Cardiovascular and Cerebrovascular Diseases,” Small 18 (2022): 2200291, 10.1002/smll.202200291. 35306751

[177] G. Y. Lee , J.‐H. Kim , K. Y. Choi , et al., “Hyaluronic Acid Nanoparticles for Active Targeting Atherosclerosis,” Biomaterials 53 (2015): 341–348, 10.1016/j.biomaterials.2015.02.089. 25890732

[178] M. A. Missinato , K. Tobita , N. Romano , J. A. Carroll , and M. Tsang , “Extracellular Component Hyaluronic Acid and Its Receptor Hmmr Are Required for Epicardial EMT during Heart Regeneration,” Cardiovascular Research 107 (2015): 487–498, 10.1093/cvr/cvv190. 26156497 PMC4540147

[179] B. Du , M. Meenu , H. Liu , and B. Xu , “A Concise Review on the Molecular Structure and Function Relationship of β‐Glucan,” International Journal of Molecular Sciences 20 (2019): 4032, 10.3390/ijms20164032. 31426608 PMC6720260

[180] K. Lee , D. Min , Y. Choi , et al., “Self‐Assembling β‐Glucan Nanomedicine for the Delivery of siRNA,” Biomedicines 8 (2020): 497, 10.3390/biomedicines8110497. 33198404 PMC7698166

[181] H. S. Goodridge , A. J. Wolf , and D. M. Underhill , “β‐Glucan Recognition by the Innate Immune System,” Immunological Reviews 230 (2009): 38–50, 10.1111/j.1600-065X.2009.00793.x. 19594628 PMC6618291

[182] G. D. Brown and S. Gordon , “A New Receptor for β‐glucans,” Nature 413 (2001): 36–37, 10.1038/35092620. 11544516

[183] G. D. Brown , “Dectin‐1: a Signalling Non‐TLR Pattern‐recognition Receptor,” Nature Reviews Immunology 6 (2006): 33–43, 10.1038/nri1745. 16341139

[184] G. Bashiri , M. S. Padilla , K. L. Swingle , S. J. Shepherd , M. J. Mitchell , and K. Wang , “Nanoparticle Protein Corona: from Structure and Function to Therapeutic Targeting,” Lab on a Chip 23 (2023): 1432–1466, 10.1039/D2LC00799A. 36655824 PMC10013352

[185] N. Bertrand , P. Grenier , M. Mahmoudi , et al., “Mechanistic Understanding of in Vivo Protein Corona Formation on Polymeric Nanoparticles and Impact on Pharmacokinetics,” Nature Communications 8 (2017): 777, 10.1038/s41467-017-00600-w. PMC5626760 28974673

[186] Y. Adachi , T. Ishii , Y. Ikeda , et al., “Characterization of β‐Glucan Recognition Site on C‐Type Lectin, Dectin 1,” Infection and Immunity 72 (2004): 4159–4171, 10.1128/IAI.72.7.4159-4171.2004. 15213161 PMC427417

[187] G. D. Brown , P. R. Taylor , D. M. Reid , et al., “Dectin‐1 Is a Major β‐Glucan Receptor on Macrophages,” The Journal of Experimental Medicine 196 (2002): 407–412, 10.1084/jem.20020470. 12163569 PMC2193936

[188] Q. Fan , R. Tao , H. Zhang , et al., “Dectin‐1 Contributes to Myocardial Ischemia/Reperfusion Injury by Regulating Macrophage Polarization and Neutrophil Infiltration,” Circulation 139 (2019): 663–678, 10.1161/CIRCULATIONAHA.118.036044. 30586706

[189] Z. Liu , W. Lian , Q. Long , et al., “Promoting Cardiac Repair through Simple Engineering of Nanoparticles with Exclusive Targeting Capability toward Myocardial Reperfusion Injury by Thermal Resistant Microfluidic Platform,” Advanced Functional Materials 32 (2022): 2204666, 10.1002/adfm.202204666.

[190] N. F. Ilahibaks , T. A. Kluiver , O. G. de Jong , et al., “Extracellular Vesicle‐Mediated Delivery of CRISPR/Cas9 ribonucleoprotein Complex Targeting Proprotein Convertase Subtilisin‐Kexin Type 9 (Pcsk9) in Primary Mouse Hepatocytes,” Journal of Extracellular Vesicles 13 (2024): 12389, 10.1002/jev2.12389. 38191764 PMC10774704

[191] S. I. van de Wakker , F. M. Meijers , J. P. G. Sluijter , and P. Vader , “Extracellular Vesicle Heterogeneity and Its Impact for Regenerative Medicine Applications,” Pharmacological Reviews 75 (2023): 1043–1061, 10.1124/pharmrev.123.000841. 37280097

[192] Y. Yuana , A. Sturk , and R. Nieuwland , “Extracellular Vesicles in Physiological and Pathological Conditions,” Blood Reviews 27 (2013): 31–39, 10.1016/j.blre.2012.12.002. 23261067

[193] M. T. Roefs , J. P. G. Sluijter , and P. Vader , “Extracellular Vesicle‐Associated Proteins in Tissue Repair,” Trends in Cell Biology 30 (2020): 990–1013, 10.1016/j.tcb.2020.09.009. 33069512

[194] C. Salomon , S. Das , U. Erdbrügger , et al., “Extracellular Vesicles and Their Emerging Roles as Cellular Messengers in Endocrinology: an Endocrine Society Scientific Statement,” Endocrine Reviews 43 (2022): 441–468, 10.1210/endrev/bnac009. 35552682 PMC10686249

[195] T. Lener , M. Gimona , L. Aigner , et al., “Applying Extracellular Vesicles Based Therapeutics in Clinical Trials—An ISEV Position Paper,” Journal of Extracellular Vesicles 4 (2015): 30087, 10.3402/jev.v4.30087. 26725829 PMC4698466

[196] M. A. Kumar , S. K. Baba , H. Q. Sadida , et al., “Extracellular Vesicles as Tools and Targets in Therapy for Diseases,” Signal Transduction and Targeted Therapy 9 (2024): 27, 10.1038/s41392-024-01735-1. 38311623 PMC10838959

[197] H. C. Verdera , J. J. Gitz‐Francois , R. M. Schiffelers , and P. Vader , “Cellular Uptake of Extracellular Vesicles Is Mediated by Clathrin‐independent Endocytosis and Macropinocytosis,” Journal of Controlled Release 266 (2017): 100–108, 10.1016/j.jconrel.2017.09.019. 28919558

[198] M. Kou , L. Huang , J. Yang , et al., “Mesenchymal Stem Cell‐derived Extracellular Vesicles for Immunomodulation and Regeneration: a next Generation Therapeutic Tool?,” Cell Death & Disease 13 (2022): 580, 10.1038/s41419-022-05034-x. 35787632 PMC9252569

[199] S.‐J. Sun , R. Wei , F. Li , S.‐Y. Liao , and H.‐F. Tse , “Mesenchymal Stromal Cell‐derived Exosomes in Cardiac Regeneration and Repair,” Stem Cell Reports 16 (2021): 1662–1673, 10.1016/j.stemcr.2021.05.003. 34115984 PMC8282428

[200] R. C. de Abreu , H. Fernandes , P. A. da Costa Martins , S. Sahoo , C. Emanueli , and L. Ferreira , “Native and Bioengineered Extracellular Vesicles for Cardiovascular Therapeutics,” Nature Reviews Cardiology 17 (2020): 685–697, 10.1038/s41569-020-0389-5. 32483304 PMC7874903

[201] J. Zhao , X. Li , J. Hu , et al., “Mesenchymal Stromal Cell‐derived Exosomes Attenuate Myocardial Ischaemia‐reperfusion Injury through miR‐182‐regulated Macrophage Polarization,” Cardiovascular Research 115 (2019): 1205–1216, 10.1093/cvr/cvz040. 30753344 PMC6529919

[202] J. Gan , X. Zhang , W. Ma , Y. Zhao , and L. Sun , “Antibacterial, Adhesive, and MSC Exosomes Encapsulated Microneedles with Spatio‐temporal Variation Functions for Diabetic Wound Healing,” Nano Today 47 (2022): 101630, 10.1016/j.nantod.2022.101630.

[203] D. Zhu , S. Liu , K. Huang , et al., “Intrapericardial Exosome Therapy Dampens Cardiac Injury via Activating Foxo3,” Circulation Research 131 (2022): E135, 10.1161/CIRCRESAHA.122.321384. 36252111 PMC9667926

[204] K. Lv , Q. Li , L. Zhang , et al., “Incorporation of Small Extracellular Vesicles in Sodium Alginate Hydrogel as a Novel Therapeutic Strategy for Myocardial Infarction,” Theranostics 9 (2019): 7403–7416, 10.7150/thno.32637. 31695776 PMC6831299

[205] Z. Zhang , J. Yang , W. Yan , Y. Li , Z. Shen , and T. Asahara , “Pretreatment of Cardiac Stem Cells with Exosomes Derived from Mesenchymal Stem Cells Enhances Myocardial Repair,” Journal of the American Heart Association 5 (2016): 002856, 10.1161/JAHA.115.002856. PMC4859399 26811168

[206] R. A. Kore , X. Wang , J. C. Henson , Z. Ding , A. Jamshidi‐Parsian , and J. L. Mehta , “Proteomic Basis of Modulation of Postischemic Fibrosis by MSC Exosomes,” American Journal of Physiology‐Regulatory, Integrative and Comparative Physiology 321 (2021): R639, 10.1152/ajpregu.00124.2021. 34431382

[207] C. J. Charles , R. R. Li , T. Yeung , et al., “Systemic Mesenchymal Stem Cell‐Derived Exosomes Reduce Myocardial Infarct Size: Characterization with MRI in a Porcine Model,” Frontiers in Cardiovascular Medicine 7 (2020), 10.3389/fcvm.2020.601990. PMC7701257 33304934

[208] R. A. Kore , X. Wang , Z. Ding , R. J. Griffin , A. J. Tackett , and J. L. Mehta , “MSC Exosome‐mediated Cardioprotection in Ischemic Mouse Heart Comparative Proteomics of Infarct and Peri‐infarct Areas,” Molecular and Cellular Biochemistry 476 (2021): 1691–1704, 10.1007/s11010-020-04029-6. 33423165 PMC8186026

[209] J. Wan , S. Lin , Z. Yu , et al., “Protective Effects of MicroRNA‐200b‐3p Encapsulated by Mesenchymal Stem Cells–Secreted Extracellular Vesicles in Myocardial Infarction via Regulating BCL2L11,” Journal of the American Heart Association 11, no. 11 (2022): 024330, 10.1161/JAHA.121.024330. PMC9238663 35699193

[210] J.‐H. Jung , X. Fu , and P. C. Yang , “Exosomes Generated from iPSC‐Derivatives,” Circulation Research 120 (2017): 407–417, 10.1161/CIRCRESAHA.116.309307. 28104773 PMC5260934

[211] Z. Gu , Z. Yin , P. Song , et al., “Safety and Biodistribution of Exosomes Derived from human Induced Pluripotent Stem Cells,” Frontiers in Bioengineering and Biotechnology 10 (2022): 949724, 10.3389/fbioe.2022.949724. 36091443 PMC9461140

[212] S. Bobis‐Wozowicz , K. Kmiotek , M. Sekula , et al., “Human Induced Pluripotent Stem Cell‐Derived Microvesicles Transmit RNAs and Proteins to Recipient Mature Heart Cells Modulating Cell Fate and Behavior,” Stem Cells 33 (2015): 2748–2761, 10.1002/stem.2078. 26031404

[213] M. Chandy , J.‐W. Rhee , M. O. Ozen , et al., “Atlas of Exosomal microRNAs Secreted from Human iPSC‐Derived Cardiac Cell Types,” Circulation 142 (2020): 1794–1796, 10.1161/CIRCULATIONAHA.120.048364. 33136510 PMC8135104

[214] S. El Andaloussi , I. Mäger , X. O. Breakefield , and M. J. A. Wood , “Extracellular Vesicles: Biology and Emerging Therapeutic Opportunities,” Nature Reviews Drug Discovery 12 (2013): 347–357, 10.1038/nrd3978. 23584393

[215] X. Wang , Y. Chen , Z. Zhao , et al., “Engineered Exosomes with Ischemic Myocardium‐Targeting Peptide for Targeted Therapy in Myocardial Infarction,” Journal of the American Heart Association 7 (2018): 008737, 10.1161/JAHA.118.008737. PMC6201471 30371236

[216] K. I. Mentkowski and J. K. Lang , “Exosomes Engineered to Express a Cardiomyocyte Binding Peptide Demonstrate Improved Cardiac Retention in Vivo,” Scientific Reports 9 (2019): 10041, 10.1038/s41598-019-46407-1. 31296886 PMC6624248

[217] A. G. E. Ibrahim , C. Li , R. Rogers , et al., “Augmenting Canonical Wnt Signalling in Therapeutically Inert Cells Converts Them into Therapeutically Potent Exosome Factories,” Nature Biomedical Engineering 3 (2019): 695–705, 10.1038/s41551-019-0448-6. PMC6736698 31451800

[218] D. Li , Y. Son , M. Jang , S. Wang , and W. Zhu , “Genome‐Wide Association Analysis of Reproductive Traits in Chinese Holstein Cattle,” Genes 15 (2023): 12, 10.3390/genes15010012. 38275594 PMC10815438

[219] M. G. Katz , J. D. Swain , J. D. White , D. Low , H. Stedman , and C. R. Bridges , “Cardiac Gene Therapy: Optimization of Gene Delivery Techniques in Vivo,” Human Gene Therapy 21 (2010): 371–380, 10.1089/hum.2009.164. 19947886 PMC2865214

[220] D. Rosenblum , N. Joshi , W. Tao , J. M. Karp , and D. Peer , “Progress and Challenges towards Targeted Delivery of Cancer Therapeutics,” Nature Communications 9 (2018): 1410, 10.1038/s41467-018-03705-y. PMC5897557 29650952

[221] M. Lundqvist , J. Stigler , G. Elia , I. Lynch , T. Cedervall , and K. A. Dawson , “Nanoparticle Size and Surface Properties Determine the Protein Corona with Possible Implications for Biological Impacts,” Proceedings of the National Academy of Sciences 105 (2008): 14265–14270, 10.1073/pnas.0805135105. PMC2567179 18809927

[222] F. S. M. Tekie , M. Hajiramezanali , P. Geramifar , et al., “Controlling Evolution of Protein Corona: a Prosperous Approach to Improve Chitosan‐based Nanoparticle Biodistribution and Half‐life,” Scientific Reports 10 (2020): 9664, 10.1038/s41598-020-66572-y. 32541900 PMC7295777

[223] J. Bejarano , M. Navarro‐Marquez , F. Morales‐Zavala , et al., “Nanoparticles for Diagnosis and Therapy of Atherosclerosis and Myocardial Infarction: Evolution toward Prospective Theranostic Approaches,” Theranostics 8 (2018): 4710–4732, 10.7150/thno.26284. 30279733 PMC6160774

[224] H. Weng , W. Zou , F. Tian , et al., “Inhalable Cardiac Targeting Peptide Modified Nanomedicine Prevents Pressure Overload Heart Failure in Male Mice,” Nature Communications 15 (2024): 6058, 10.1038/s41467-024-50312-1. PMC11258261 39025877

[225] Y. Chen , S. Liu , Y. Liang , et al., “Single Dose of Intravenous miR199a‐5p Delivery Targeting Ischemic Heart for Long‐term Repair of Myocardial Infarction,” Nature Communications 15 (2024): 5565, 10.1038/s41467-024-49901-x. PMC11219733 38956062

[226] T. Tashima , “Nanoparticle‐Based Targeted Drug Delivery Methods for Heart‐Specific Distribution in Cardiovascular Therapy,” Pharmaceutics 17 (2025): 1365, 10.3390/pharmaceutics17111365. 41304704 PMC12655773

[227] K. T. Butler , D. W. Davies , H. Cartwright , O. Isayev , and A. Walsh , “Machine Learning for Molecular and Materials Science,” Nature 559 (2018): 547–555, 10.1038/s41586-018-0337-2. 30046072

[228] B. Li , I. O. Raji , A. G. R. Gordon , et al., “Accelerating Ionizable Lipid Discovery for mRNA Delivery Using Machine Learning and Combinatorial Chemistry,” Nature Materials 23 (2024): 1002–1008, 10.1038/s41563-024-01867-3. 38740955

[229] B. J. Shields , J. Stevens , J. Li , et al., “Bayesian Reaction Optimization as a Tool for Chemical Synthesis,” Nature 590 (2021): 89–96, 10.1038/s41586-021-03213-y. 33536653

[230] C. B. Wahl , M. Aykol , J. H. Swisher , J. H. Montoya , S. K. Suram , and C. A. Mirkin , “Machine Learning–accelerated Design and Synthesis of Polyelemental Heterostructures,” Science Advances 7 (2021): abj5505, 10.1126/sciadv.abj5505. PMC8694626 34936439

[231] L. Rao , Y. Yuan , X. Shen , G. Yu , and X. Chen , “Designing Nanotheranostics with Machine Learning,” Nature Nanotechnology 19 (2024): 1769–1781, 10.1038/s41565-024-01753-8. 39362960

[232] N. Boehnke , J. P. Straehla , H. C. Safford , et al., “Massively Parallel Pooled Screening Reveals Genomic Determinants of Nanoparticle Delivery,” Science 377 (2022): abm5551, 10.1126/science.abm5551. PMC10249039 35862544

[233] M. Zhu , J. Zhuang , Z. Li , et al., “Machine‐learning‐assisted Single‐vessel Analysis of Nanoparticle Permeability in Tumour Vasculatures,” Nature Nanotechnology 18 (2023): 657–666, 10.1038/s41565-023-01323-4. 36781994

[234] S. Dhoble , T.‐H. Wu , and Kenry , “Decoding Nanomaterial‐Biosystem Interactions through Machine Learning,” Angewandte Chemie International Edition 63 (2024): 202318380, 10.1002/anie.202318380. 38687554

[235] J. S. Smith , D. Bedrov , and G. D. Smith , “A Molecular Dynamics Simulation Study of Nanoparticle Interactions in a Model Polymer‐nanoparticle Composite,” Composites Science and Technology 63 (2003): 1599–1605, 10.1016/S0266-3538(03)00061-7.

[236] X. Zhang , G. Ma , and W. Wei , “Simulation of Nanoparticles Interacting with a Cell Membrane: Probing the Structural Basis and Potential Biomedical Application,” NPG Asia Materials 13 (2021): 52, 10.1038/s41427-021-00320-0.

[237] M. H. Teplensky , M. Fantham , C. Poudel , et al., “A Highly Porous Metal‐Organic Framework System to Deliver Payloads for Gene Knockdown,” Chemistry (Weinheim An Der Bergstrasse, Germany) 5 (2019): 2926–2941, 10.1016/j.chempr.2019.08.015.

[238] Y. Zhang , J. L. Y. Wu , J. Lazarovits , and W. C. W. Chan , “An Analysis of the Binding Function and Structural Organization of the Protein Corona,” Journal of the American Chemical Society 142 (2020): 8827–8836, 10.1021/jacs.0c01853. 32293877

[239] S. Hamilton and B. R. Kingston , “Applying Artificial Intelligence and Computational Modeling to Nanomedicine,” Current Opinion in Biotechnology 85 (2024): 103043, 10.1016/j.copbio.2023.103043. 38091874

[240] P. Sarker , M. S. J. Sajib , X. Tao , and T. Wei , “Multiscale Simulation of Protein Corona Formation on Silver Nanoparticles: Study of Ovispirin‐1 Peptide Adsorption,” The Journal of Physical Chemistry B 126 (2022): 601–608, 10.1021/acs.jpcb.1c08267. 35026946

[241] B. Kang , T. Opatz , K. Landfester , and F. R. Wurm , “Carbohydrate Nanocarriers in Biomedical Applications: Functionalization and Construction,” Chemical Society Reviews 44 (2015): 8301–8325, 10.1039/C5CS00092K. 26278884

[242] C. Chen , Z. Yaari , E. Apfelbaum , P. Grodzinski , Y. Shamay , and D. A. Heller , “Merging Data Curation and Machine Learning to Improve Nanomedicines,” Advanced Drug Delivery Reviews 183 (2022): 114172, 10.1016/j.addr.2022.114172. 35189266 PMC9233944

[243] Y.‐Q. Chen , M.‐D. Xue , J.‐L. Li , D. Huo , H.‐M. Ding , and Y. Ma , “Uncovering the Importance of Ligand Mobility on Cellular Uptake of Nanoparticles: Insights from Experimental, Computational, and Theoretical Investigations,” ACS Nano 18 (2024): 6463–6476, 10.1021/acsnano.3c11982. 38346263

